# A systematic review of adolescent alcohol‐related harm trends in high‐income countries with declines in adolescent consumption

**DOI:** 10.1111/add.70026

**Published:** 2025-03-05

**Authors:** Emma Vieira, Nicholas Taylor, Abigail Stevely, Amy Pennay, Jonas Raninen, John Holmes, Rakhi Vashishtha, Michael Livingston

**Affiliations:** ^1^ National Drug Research Institute Curtin University Perth Western Australia Australia; ^2^ Burnet Institute Melbourne Victoria Australia; ^3^ Sheffield Addictions Research Group, School of Medicine and Population Health University of Sheffield Sheffield UK; ^4^ Centre for Alcohol Policy Research La Trobe University Melbourne Victoria Australia; ^5^ Department of Clinical Neuroscience Karolinska Institutet Stockholm Sweden; ^6^ Programme in Health services and Systems Research Duke‐NUS Medical School Singapore

**Keywords:** adolescents, alcohol, alcohol‐related harm, sex‐based differences, systematic review, trends

## Abstract

**Background and Aims:**

Adolescent alcohol consumption decreased in high‐income countries during the 2000s and 2010s. While evidence for declining consumption is clear, there has been less research tracking trends in alcohol‐related harms. This article reviewed trends in adolescent alcohol‐related harms in high‐income countries where a decline in consumption had occurred and investigated sex‐based differences in trends.

**Methods:**

The databases Medline, CINAHL, Scopus and PubMed were systematically searched, with grey literature searches also conducted. Studies were included if they reported on harm rates between 2005 and 2019 for adolescents (10–19 years) from countries where a reduction in adolescent drinking occurred. Health‐system based measures of alcohol‐related harm were used (e.g. hospital admissions or mortality data). Search terms included alcohol, adolescents, alcohol‐related harms, trends or synonyms. Risk of bias was assessed, primary screening was conducted by one author with checks by another, and data extraction was completed by three authors with accuracy checks conducted. The results are presented via narrative synthesis.

**Results:**

Systematic searches resulted in 1311 results. A total of 18 systematic search and 23 grey literature sources were included. For many countries, alcohol‐related harms have decreased since 2005, following trends in declining consumption. This evidence was strongest in Anglosphere countries, where eight of thirteen records (62%) indicated declines, followed by North America, where declines were present in four of eleven records (36%). Trends from mainland Europe were contradictory, with only four of thirteen (31%) indicating decreases in harms. Increases in harms for some female and student populations were reported in some jurisdictions.

**Conclusions:**

Alcohol‐related harms for young people have generally declined in countries where youth drinking has fallen, although the declines in harm have been smaller than the declines in drinking. Declines in alcohol‐related harm were strongest in the United Kingdom, Australia, New Zealand and Ireland, followed by North America.

## INTRODUCTION

Alcohol consumption is one of the leading causes of preventable death, illness and injury worldwide [1, 2]. In many high‐income countries, initiation of alcohol use commonly occurs during adolescence, and earlier initiation of alcohol consumption is associated with heavier use in adulthood and higher levels of harms [3]. Evidence suggests that adolescent alcohol consumption has decreased, mostly in high‐income countries from the 2000s to 2010s [4, 5]. Both the prevalence of drinking and levels of consumption across multiple measures appear to be declining across most socio‐demographic groups [6, 7, 8, 9], barring some higher risk subgroups [4, 7, 10]. No single factor appears to drive the decline, but changing influences beyond alcohol, such as changing dispositions to risk, increases in parental control and changes in leisure activities and self‐governance, appear to be important contributors [11, 12, 13, 14, 15]. An investigation of this decline in adolescent consumption indicated that it largely occurred from the early 2000s onward in high‐income countries, beginning in North America before spreading to parts of Europe and to Australasia [5]. Although that study focused on past‐month consumption trends until approximately the mid‐2010s, updated evidence from this time broadly indicates a continuing decline in adolescent past‐month alcohol consumption until 2019 (see Table S1).

Although evidence consistently indicates adolescent alcohol consumption is decreasing, there has been less research tracking trends in alcohol‐related harms among young people. Given the reduction in adolescent consumption throughout many high‐income countries, associated harms should also be declining at a comparable rate. However, research from more than 10 years ago identified divergences between consumption and harm trends for adults, with various alcohol‐related harms (including health, mortality, crime and traffic‐related measures) rapidly increasing in the early 2000s over a period of stable or declining consumption [16, 17]. More recent evidence from Sweden suggests that trends in both alcohol‐related consumption and harms in the form of self‐reported alcohol‐related problems and medical care events have been declining in tandem from the 2000s onward for adolescents [18, 19]. To date, it is unclear whether harm is declining in line with consumption in many countries, or whether there are differences in trends across countries.

Further, given some evidence that drinking declines have been larger for boys than girls [9], it is possible that alcohol‐related harm trends will have varied between boys and girls. Many countries have also seen a convergence between adolescent boys' and girls' drinking over the years (in measures of both frequency and quantity of consumption), with sex‐based differences now non‐existent or less obvious than before, or in some countries, girls having overtaken boys in some measures of consumption [20, 21]. However, as with consumption, there is little investigation understanding how alcohol‐related harms may be impacted by sex for adolescents in countries where consumption has declined.

This article is the first to review trends in adolescent alcohol‐related harms in countries where adolescent consumption has declined. We use health‐system based measures of harm and focus our search on high‐income countries that have reported reductions in adolescent consumption since the early 2000s, because these countries should have experienced the most substantial and, therefore, detectable reductions in alcohol‐related harms. Our primary aim was to determine whether adolescent alcohol‐related harms have decreased over the same period as the decline in adolescent alcohol consumption in 19 countries. Differences in trends by sex were considered as a secondary outcome.

## METHODS

The review was conducted using the Preferred Reporting Items for Systematic reviews and Meta‐Analyses (PRISMA) guidelines [22]. The protocol for this review was registered on PROSPERO (CRD42023432878). The review was conducted in a two‐stage process. First, a systematic search of peer‐reviewed literature was completed. A search of the grey literature was conducted in the second stage, because it was expected that much of the relevant data would be available only in government monitoring and reporting systems.

### Eligibility criteria

#### Population

Studies must include data on adolescents between 10‐ and 19‐years old, either as an age group within this range or as a young adult group up to age 34 that overlaps with this range (e.g. age groups of 18–24 or 15–34 included). Including studies with an overlap of adolescent and young adult ages was necessary to ensure data was captured for as many countries as possible, as health population statistics have varying methods of grouping young people into age brackets. This method has been used effectively in previous work [23]. Whole population data was excluded.

We have based our country eligibility criteria on data from high‐income countries reported by Vashishtha *et al*. [5] and included countries that they reported as having at least a 30% reduction in the prevalence of adolescent drinking since the early 2000s. Territories and other regions related to those countries (e.g. Faroe Islands, Guam, *etc*.) were not included in the search. Therefore, there were 19 eligible countries that reported past‐month declines in adolescent consumption ranging from 30% to 84%, with the specific countries, consumption declines and time periods shown in Table 1.

**TABLE 1:** Table based on the results of Vashishtha *et al*. [5], indicating high‐income countries reporting a decline of at least 30% in adolescent past‐month drinking

Country	Time period^a^	Percentage decline (%)^b^
Australia	2002–2017	44.9
Austria	2005/2006–2013/2014	36.6
Belgium	2005/2006–2013/2014	37.5 (Flemish); 38.2 (French)
Canada	2001/2002–2013/2014	38.4
Estonia	1999–2015	38.7
Finland	1999–2015	47.5
Germany	2001/2002–2013/2014	30.4
Iceland	1995–2015	83.9
Ireland	1997/1998–2013/2014	64.4
Lithuania	2003–2015	55.8
The Netherlands	2005/2006–2013/2014	41.1
New Zealand	2001–2012	43.0
Norway	1999–2015	60.0
Portugal	2007–2015	30.0
Spain	2009/2010–2013/2014	35.7
Sweden	1999–2015	53.6
Switzerland	2001/2002–2013/2014	44.9
United Kingdom	2001/2002–2013/2014	46.2
United States of America	1999–2017	40.4

^a^ Time period is noted here as the first year of the decline until the most recent data available.

^b^ Percentage change is calculated from the first year of the decline until the most recent data available.

#### Intervention

As this review focuses on time trends, the intervention is time itself. The search was limited to studies that included data from after 2005, reflecting the period in which youth drinking has declined [4, 5, 6, 24], and ensuring we captured a period where all countries included in the review had experienced declines in consumption. For the grey literature search, studies with data before 2005 were included where this data also encompassed our time period of interest, specifically for countries where no other data was available. We did not include data from 2020 onward to reduce the potential impact of coronavirus disease 2019 on our results (e.g. Foundation for Alcohol Research and Education and White *et al*. [25, 26]). Studies were also required to have data for at least two timepoints to be included.

#### Outcome

Studies must have recorded a health‐system based measure of alcohol‐related harm to be eligible for inclusion. Alcohol‐related harms are defined as harms to the self from alcohol (as opposed to harms because of others' drinking). These harms included, but were not limited to, emergency department (ED) presentations or mortality data directly linked to alcohol use, as indicated by specific International Classification of Diseases (ICD) codes, assessments of alcohol involvement by clinicians, blood alcohol content measurement or other relevant criteria (see results section for further information). The World Health Organization (WHO) rates health systems data from high‐income countries as a generally robust way to measure acute alcohol‐related harms [1]. This outcome also reduces the potential impact of self‐report and sampling biases that may impact survey data.

Any journal articles not written in English were excluded. When conducting the grey literature searches, Google Translate was used where authors encountered text in languages other than English.

### Search strategy

The detailed search strategy can be found in Material S1. The databases Medline, CINAHL, Scopus and PubMed were systematically searched using terms relating to adolescents, alcohol‐related harms, health‐system based measurements of harm and trend‐related terms (Table S2). The grey literature search focused on data that was accessible to the general public via Google searches, with E.V., N.T. and M.L. screening the first 10 pages of results, following the method outlined by Godin *et al*. [27]. Experts were contacted by M.L. to assist in identifying grey literature for specific countries (see Table 2 for results) [27]. Additionally, backward snowballing was conducted.

**TABLE 2:** Methods of searching for grey literature (*n* = 23) and results of each

Method of searching	No. of sources identified	Countries
Contacts experts^a^	7	Canada, Germany, Lithuania, New Zealand, Sweden, UK
Backward snowballing	3	Canada, USA
Manual Google searches	12	Australia, Austria, Belgium, Canada, Estonia, Ireland, The Netherlands, Spain, Switzerland, USA
Peer review addition	1	Finland

Abbreviations: UK, United Kingdom; USA, United States of America

^a^ Colleagues in Finland and Norway were also contacted, but no results were returned.

### Screening and data extraction

Screening and data extraction details are available in Material S1. Results from the systematic search were screened in Endnote by E.V., with N.T. randomly assigned a 10% subset of all records to check this screening. Full agreement between the two authors was found. Full‐text screening and data extraction was conducted in Covidence or Microsoft Excel. Extraction was split between E.V., N.T. and M.L., with a secondary researcher checking and confirming each extraction—any disagreements were discussed and decided on by the senior author (M.L.) The full extraction variables can be found in Table 3, which included basic source details (e.g. author, publication year), country of interest, population age group, time period of study, origin/source of data, outcome measurement, key results and any biases/limitations of the data.

**TABLE 3:** Data extraction form

Information extracted		Example data extractions
Data information	Title	Article title example: Alcohol‐related trauma presentations among older teenagers [77] Database title example: hospital admissions and patients (Ziekenhuisopnamen en‐patiënten) [55]
	Author	Article author example: Green *et al*. [73] Database author example: Public Health England [68]
	Year of publication	2022
	Source type (e.g. journal article, database, report, etc.)	Downloadable dataset
Data characteristics	Country	Belgium
	Population ages, y	16–19
	Time period of study	2008–2019
	Aim or summary	Database example: the Federal Health Monitoring System. ‘DESTATIS’ website; can search by topic (such as F10 or T51) to generate customisable tables using health care data [87]
	Origins/sources of the data	The data is from the AIHW's NHMD [80]
	Outcome measured – general (summary of main outcome)	Cases of adolescents treated with alcohol intoxication from a department of paediatrics [62]
	Outcome measured – specific (e.g. inclusion criteria, ICD codes, *etc*.)	Specific criteria: 1. Patients admitted to a department of paediatrics in 2007, 2008 or 2009 2. Patients age 11‐ to 17‐years‐old 3. Patients had a BAC >0 g/L 4. Patients admitted because of impaired consciousness [74]
Results	Any relevant demographics/statistics approaches	3443 students <20 years old appeared across the 5 years [61]
	Key results	Decrease in ED visits over the time period, from a rate of 31.64 per 100 000 (*n* = 2816) in 2007, to 13.12 per 100 000 (*n* = 1289) in 2019 [60]
	Sex‐specific results (if applicable)	Males decreased: *n* = 4152 to 2959 from 2010/2011–2016/2017 Females remained stable: *n* = 2120 to 2011 from 2010/2011–2016/2017 [80]
	Direction of trend in main outcome (i.e. increase, decrease, stability, or mixed)	Mixed
	Details on direction of trend (e.g. how is it mixed)	Males decrease, females stable
	Proportion increase/decreases in percentage	17% increase in the rates of female ED visits per 100 000 population between 2008 and 2019 16% decrease in the rates of male ED visits per 100 000 population between 2008 and 2019 [59]
Other information	Biases/limitations in the data (e.g. data beyond inclusion criteria)	Age group 15‐ to 34‐year‐olds
	Any further notes	Google translate used for column headings

Abbreviations: AIHW, Australian Institute of Health and Welfare; BAC, blood alcohol content; ED, emergency department; NHMD, National Hospital Morbidity Dataset.

### Quality assessment tool

Risk of bias was assessed using the tool created by Hoy *et al*. [28] to assess bias in population‐based prevalence studies. The tool contains 10 items that are ranked as either low or high risk of bias, with a summary item for overall risk of bias reported as either low, moderate or high [28]. Studies were reported as having an overall low risk of bias if two or less items were scored ‘high risk’, moderate if three or four items were scored ‘high risk’ and high if five or more items were scored ‘high risk.’

### Synthesis of results

The primary outcome was any change in alcohol‐related harms between the start and end of each study's time period, calculated as a basic percentage change. Where subgroup data were available, any changes based on sex were also examined. A threshold for determining change of 10% relative from baseline was decided by the authors before examining the papers, with any percentage change of 10% or less indicating a stability in rates of alcohol‐related harm. This amount was chosen given that we include countries where a minimum 30% decline in consumption occurred [5], we would expect at least a 10% decline in alcohol‐related harms to indicate meaningful change. The implications of this choice are discussed in the limitations section. Sensitivity analyses were conducted and are reported below in the results section.

## RESULTS

### Description of included studies

**FIGURE 1:**
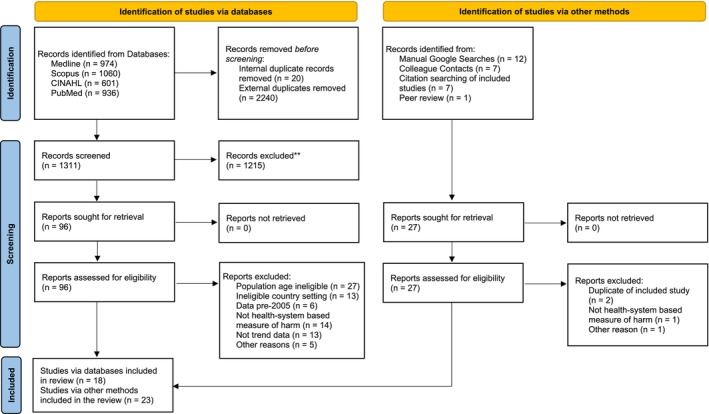
Preferred Reporting Items for Systematic Reviews and Meta‐Analyses (PRISMA) 2020 flow diagram for new systematic reviews that included searches of databases, registers and other sources.

The PRISMA diagram is displayed in Figure 1. The initial systematic search resulted in 1311 studies, of which 96 were progressed to full‐text screening. After full text screening, a total of 18 sources remained from the systematic search, including four additional sources identified during the final searches [18, 29, 30, 31]. There were 23 sources identified through other methods in the grey literature search, with a total of 41 sources included in this review. It should be noted that the data for Estonia [32, 33, 34, 35, 36, 37] and Finland [38, 39, 40, 41, 42, 43, 44, 45, 46, 47, 48, 49, 50, 51, 52] are comprised of several Yearbook reports that E.V. compiled into a single dataset to consider trends over time. Data for these countries are presented as a single data source throughout. Five of the included sources required partial translation using Google Translate [53, 54, 55, 56, 57].

Although there are 41 sources, the results have been split into 46 records. Three sources reported multiple distinct outcomes that were split into separate records for ease of interpretation. The first study [58] investigated Australia and the United Kingdom separately, with both countries indicating different directions of alcohol‐related harm rates. Therefore, one record considered only the Australian results, whereas the other considered only the results from the United Kingdom. The remaining two studies [59, 60] were split into separate records as they reported two different, but equally relevant, harm outcome measurements: hospital admissions and ED presentations data. For each of these studies, one record considers the ED presentations only, whereas the other reports only on hospital admissions. One source included in the final updated search reported individual data for every eligible country and has been split into three records to account for the three geographic areas reported on in this review [29]. The specific results for each country are detailed in Table S3 and briefly within the results tables below.

Data for all 19 eligible countries were identified. Of the total 46 records, 15 reported data from North America, 15 from other Anglosphere countries and the remaining 16 reported data from mainland Europe (see Table 4). With regards to harm measurement, most records (52%; *n* = 24) reported on hospital admissions data, followed by ED presentations (26%; *n* = 12). Population ages within the included records were usually around the 10 to 19 range, but grey literature included populations as young as 0 and up to 34 years of age. The 25 grey literature records were mostly raw data accessed through either an on‐line interactive database or downloadable dataset (56%; *n* = 14), with a further five records of journal articles (20%) and six on‐line reports (24%).

**TABLE 4:** Country distribution of *n* = 46 records

North America (*n* = 15 records)	Anglosphere countries (*n* = 15 records)	Mainland Europe (*n* = 16 records)
United States of America (*n* = 10) [29, 30, 31, 61, 62, 63, 64, 65, 66, 67]	United Kingdom (*n* = 8) [29, 58, 68, 69, 70, 71, 72, 73]	The Netherlands (*n* = 4) [29, 55, 74, 75]
Canada (*n* = 6) [29, 59, 60, 76]	Australia (*n* = 6) [29, 58, 77, 78, 79, 80]	Belgium (*n* = 3) [29, 81, 82]
	Ireland (*n* = 2) [29, 83]	Switzerland (*n* = 3) [29, 56, 84]
	New Zealand (*n* = 2) [29, 85]	Sweden (*n* = 3) [18, 29, 86]
		Austria (*n* = 2) [29, 54]
		Germany (*n* = 2) [29, 87]
		Lithuania (*n* = 2) [29, 57]
		Spain (*n* = 2) [29, 53]
		Estonia (*n* = 2) [29, 32, 33, 34, 35, 36, 37]
		Finland (*n* = 2) [29, 38, 39, 40, 41, 42, 43, 44, 45, 46, 47, 48, 49, 50, 51, 52]
		Iceland (*n* = 1) [29]
		Norway (*n* = 1) [29]
		Portugal (*n* = 1) [29]

*Note*: Column totals do not add to record totals as one source contains global country‐specific results [29], but this source is only split into three records for ease of interpretation.

Information regarding the nine records with a shorter timespan (5 years and under) are considered separately and can be found in Table S4. A summary table of the data extracted from records with timespans over 5 years can be found in Table 5. Information regarding the specific methods of harm measurement for each of the 41 sources can be found in Table S5.

**TABLE 5:** Summary of data extraction for all records (n = 37) with time spans over 5 years

First author, publication year OR data summary	Country	Population age, y	Study time period	Harm measurement	Data sources/origins
Trefan, 2019 [71]	UK: Wales	10–17	2006–2011	Alcohol‐related hospital admissions, according to ICD‐10 codes	The Patient Episode Database for Wales—demographic and clinical data on all inpatient and day admissions in NHS Wales hospitals and for all Welsh residents treated in England; population estimates are from the Public Health Wales Observatory
Green, 2017 [73]	UK: England	10–19	2002/2003–2013/2014^a^	Alcohol‐related hospital admissions, according to ICD‐10 codes. Admissions could be acute or chronic, and wholly or partially attributable to alcohol	Hospital Episode Statistics—routinely collected administrative data recording any hospital activity in England
Mitra, 2023 [77]	Australia	16–19	2008–2019	Presentation to a major emergency and trauma centre after an acute injury with a BAC >0.0 g/100 mL	The Alfred Hospital Trauma Registry—prospectively records pre‐hospital and hospital data chronicling trauma activity
Moise, 2019 [62]	US	10–19	2006–2010 compared to 2011–2015	Cases of adolescents with traumatic unintentional injury hospitalisations with BAC >0.0 g/100 mL	Illinois State Trauma Registry—mandatory reporting database of all presentations to level 1 and level 2 trauma centres, which include approximately a third of all hospitals in Chicago
Ngo, 2018 [61]	US	<20	2009/2010–2014/2015	ED presentations at a college because of alcohol intoxication, according to ICD‐9 codes	Data linked the ED Patients Registration System, the University's Student Information System, and the ED Clinical Data Repository
O'Donnell, 2017 [58]	UK^b^: England	13–17	1997–2012^a^	Hospital admissions with alcohol‐related injuries, according to ICD‐10 or T codes	NHS in England
Panken, 2022 [81]	Belgium	10–19	2008–2019	Alcohol‐related ED admissions with a BAC ≥0.1 g/L	Data from the tertiary referral academic teaching hospital University Hospital Leuven and the regional hospital Heilig Hart Leuven
Sims, 2021 [78]	Australia	12–24^a^	2005–2017	Alcohol‐related ED and hospital presentations, according to ICD‐10 codes, symptom codes, diagnosis discharge text and presenting complaint text	The Western Australian ED Data Collection, linked to the Hospital Morbidity Data Collection (by the WA Department of Health)—indicating all episodes of care for patients in all public and private hospitals in WA
Tyrrell, 2018 [70]	UK: England	10–14	1998–2014^a^	Medically attended alcohol poisonings, according to ICD‐10 or read codes	The Clinical Practice Research Datalink, linked to Hospital Episodes Statistics data and Office for National Statistics mortality data—providing hospital admissions, mortality and medical records data
Wicki, 2020 [84]	Switzerland	10–19	2010–2016	Proportion of hospitalisations for alcohol intoxication per 1000 overall admissions, according to ICD‐10 codes	The Swiss Hospital Statistics—mandatory recording of diagnoses for all in‐patients in Switzerland
Stafström, 2023 [18]	Sweden	15–19	2000–2021^a^	Alcohol‐related diagnoses through either in‐patient or specialised out‐patient care, according to ICD‐10 codes	The National Board of Health and Welfare—with estimates from medical record systems
Danpanichkul *et al*., 2024 [29]	Global^c^	15–19	2000–2019	Rates of chronic liver disease and cirrhosis from alcohol consumption	Data from the GBD study 2019—this dataset is compiled by the Institute for Health Metrics and Evaluation, aggregating data from multiple primary sources; the majority of this data is sourced from vital registration data provided by WHO; ICD‐10 codes are used to identify alcohol‐related cases of chronic liver disease and cirrhosis
On‐line database titled: Statistical Database, Diagnoses [86]	Sweden	10–19	2008–2019	Patients with a principal diagnosis relating to alcohol from hospital in‐patient admissions or outpatient non‐primary care	The National Board of Health and Welfare's National Patient Register
Downloadable dataset, page titled: Alcohol‐related harm data [85]	New Zealand	0–14 15–24	2005–2019	Hospital discharges for conditions wholly attributable to alcohol use	The National Minimum Dataset, provided by the Ministry of Health and the Environmental Health Intelligence New Zealand
On‐line database titled: Diagnostic data of the hospitals starting from 2000 [87]	Germany	10–14 15–19	2005–2019	Patients who were discharged from inpatient treatment in a hospital with a diagnosis of the F10.0 (mental and behavioural disorders because of use of alcohol) ICD‐10 code	The Federal Health Monitoring System's healthcare data
On‐line database titled: Local Alcohol Profiles for England [68]	UK: England	Under 18	2006/2007–2018/2019	Admission to hospitals where the primary or any secondary diagnoses are a wholly attributable alcohol‐specific condition	The Hospital Episode Statistics for admissions data and the Office for National Statistics for mid‐year population estimates, by Public Health England and the Office for Health Improvement and Disparities
Downloadable dataset, page titled: Alcohol related hospital statistics [69]	UK: Scotland	Under 15 15–19	2005/2006–2018/2019	Distinct alcohol‐related hospital admissions	Scottish Morbidity Records SMR01 (general acute inpatient and day case hospital activity) and SMR02 (psychiatric inpatient and day case hospital activity), by Public Health Scotland
On‐line report titled: Alcohol Market, Consumption and Harms in Estonia – Yearbook (Alkoholi turg, tarbimine ja kahjud Eestis – Aastaraamat) [32, 33, 34, 35, 36, 37]	Estonia	16–20	2013–2019	Patients who turned to a specialist or family doctor because of alcohol‐related diseases	Estonian Health Insurance Fund, published by the Estonian Institute of Economic Research
On‐line report titled: Mortality Attributable to Alcohol in Spain (Mortalidad Atribuible al Alcohol en España) [53]	Spain	15–34	2001–2009 compared to 2010–2017	Mortality rates attributable to alcohol for excessive or light/moderate drinkers	The mortality registry of the National Institute of Statistics; published by the Ministry of Health, Government Delegation for the National Drug Plan
On‐line report titled: Alcohol Consumption, Alcohol‐related Harm and Alcohol Policy in Ireland [83]	Ireland	0–17	1995 compared to 2018	Hospital discharges for wholly alcohol‐attributable conditions	The Hospital In‐Patient Enquiry Scheme, with data published by the Health Research Board
Downloadable dataset, page titled: Hospital Discharges from Acute Hospitals (Spitalsentlassungen aus Krankenanstalten) [54]	Austria	0–14	2005–2019	Numbers of hospital discharges for the F10.0 (mental and behavioural disorders because of use of alcohol) ICD‐10 code	Statistics Austria, based on the hospital statistics of the Ministry of Health
Smith, 2023 [59]	Canada	15–34	2008–2019	Hospitalisations for wholly or partially attributable alcohol conditions^d^	Discharge Abstract Database and Ontario Mental Health Reporting System, with data extracted using the Ontario Ministry of Health's Intelli‐HEALTH tool
Smith, 2023 [59]	Canada	15–34	2008–2019	ED visits for wholly or partially attributable alcohol conditions^d^	National Ambulatory Care and Reporting System, with data extracted using the Ontario Ministry of Health's Intelli‐HEALTH tool
Myran, 2019 [76]	Canada	10–18	2005–2016	ED visits with alcohol being listed as a main reason or having contributed to the visit	National Ambulatory Care and Reporting System
White, 2018 [64]	US	12–17	2006–2014	Hospital‐based ED visits related to all (acute or chronic) alcohol consumption	The Nationwide ED Sample—the largest existing dataset on ED visits in the US
On‐line report titled: trends in alcohol‐related morbidity among community hospital discharges [65]	US	12–20	2005–2015	Hospital discharges with principal (first‐listed) alcohol‐related diagnoses	The National Inpatient Sample—the largest publicly available inpatient care database in the US; published by the NIAAA
On‐line database titled: hospital admissions and patients (Ziekenhuisopnamen en‐patiënten) [55]	The Netherlands	1–20	2013–2019	Hospital admissions with a diagnosis of the F10.0 (mental and behavioural disorders because of use of alcohol) ICD‐10 code	The National Basic Hospital Care Register, provided by the Central Bureau of Statistics (Central Bureau voor de Statistiek)
On‐line database titled: patients in hospitals by age class, sex and diagnostic group (patients dans les hôpitaux selon la classe l'âge, le sexe et le groupe de diagnostic) [56]	Switzerland	10–14 15–19	2005–2019	Hospital admissions with a diagnosis of the F10.0 (mental and behavioural disorders because of use of alcohol) ICD‐10 code	The Hospital Medical Statistics and Federal Statistical Office of Switzerland
On‐line database titled: Canadian Substance Use Costs and Harms [60]	Canada	0–14	2007–2019	Rates of hospital admissions with diagnoses that are wholly or partially attributable to alcohol, according to the ICD‐10^d^	The Canadian Substance Use Costs and Harms Scientific Working Group
On‐line database titled: Canadian Substance Use Costs and Harms [60]	Canada	0–14	2007–2019	Rates of ED presentations with diagnoses that are wholly or partially attributable to alcohol, according to the ICD‐10^d^	The Canadian Substance Use Costs and Harms Scientific Working Group
On‐line database titled: Australian alcohol‐attributable harm visualisation tool [79]	Australia	15–34	2011–2019	Rates of wholly alcohol‐attributable hospitalisations according to ICD‐10 codes	The National Hospital Morbidity Database, provided by the Australian Institute of Health and Welfare. Data from NAIP
Downloadable dataset, page titled: alcohol‐related injury: hospitalisations and deaths, 2019–20 [80]	Australia	15–24	2010/2011–2016/2017	No. of alcohol‐related injury hospitalisations, defined as a principal diagnosis of injury and additional diagnosis relating to alcohol use or vice versa, according to ICD‐10 codes	The National Hospital Morbidity Database, provided by the Australian Institute of Health and Welfare
Downloadable dataset, page titled: national estimates of drug‐related emergency department visits, 2004–2011—all visits [67]	US	12–17 18–20 Under 21	2005–2011	Estimates of alcohol‐only related ED visits, according to the DAWN drug vocabulary and classification system to record, code and classify substances	The Centre for Behavioural Health Statistics and Quality, provided by the Drug Abuse Warning Network
On‐line database titled: prevalence—number of ill people [57]	Lithuania	10–19	2014–2019	Prevalence rates of those diagnosed with F10.0 (mental and behavioural disorders because of use of alcohol) ICD‐10 code, including on death certificates	The Compulsory Health Insurance Fund Information System and the Register of Causes of Death
On‐line report titled: Yearbook of Alcohol and Drug Statistics (Päihdetilastollinen vuosikirja; Alkoholi ja huumeet) [38, 39, 40, 41, 42, 43, 44, 45, 46, 47, 48, 49, 50, 51, 52]	Finland	0–14 15–19	2005–2019	No. of hospital inpatient care periods with a primary diagnosis of alcohol, according to ICD‐9/10 codes	The National Institute for Health and Welfare's Care Register for Health Care

Abbreviations: BAC, blood alcohol content; DAWN, drug abuse warning network; ED, emergency department; GBD, Global Burden of Disease; ICD, International Classification of Diseases; NHS, National Health Service; NIAA, National Institute on Alcohol Abuse and Alcoholism; NAIP, National Alcohol Indicators Project; UK, United Kingdom; US, United States; WA, Western Australia; WHO, World Health Organisation.

^a^ Some variables extend beyond the original exclusion criteria but were still chosen for inclusion. Further information available in Quality Assessment section.

^b^ Source has been split into two records to reflect differing results for separate countries (Australia and UK).

^c^ Source is usually three records to reflect differing geographical areas, but one row used as all summary information identical.

^d^ Source has been duplicated into two records to reflect differing results for separate hospital admissions and ED results.

### Quality assessment results

The results of the quality assessment can be found in Table S6. The 41 sources were randomly assigned for quality assessment by E.V. (39%; *n* = 16), N.T. (29%; *n* = 12) and M.L. (32%; *n* = 13) for quality assessment. Blind checking was randomly assigned among the three assessors, with a total of 22 disagreements in scoring (53%) discussed together and decided on by the three assessors. The majority of included studies (90%; *n* = 37) had an overall low risk of bias score. Four sources (10%) had a moderate risk of bias score, whereas none had a high risk of bias score.

Many of the grey literature sources (71%; *n* = 17) included variables that went beyond the boundaries of the pre‐registered eligibility criteria, mostly because of population age groups that included additional ages outside the 10 to 19 range (e.g. a 15–24 age group). These cases are highlighted in Table S7, but the records were still included in the analysis to ensure data from under‐researched countries were captured.

### Sensitivity analysis

The use of a 10% threshold to determine meaningful change is essentially arbitrary, so we assessed the sensitivity of our results to this decision using the records with timespans over 5 years. If this threshold was moved to 15%, only three records would be impacted. Two records would become mixed trends (from a decrease and an increase) and one would become stable from mixed. If the threshold was moved to 5%, seven records would be impacted. Three records would become decreasing (two from mixed and one from stable), one would become increasing from stable, two records would become mixed from stable and one mixed would change how it is mixed. Given no clear trend emerged from adjusting this threshold, the original was maintained.

### Presentation of results

We chose to present our results based on geographic areas (North America, other Anglosphere countries and mainland Europe) to reflect the way Vashishtha *et al*. [5] reported consumption findings, as we compare these consumption trends to our own reported harm trends throughout. However, the organisation of countries in our study differ from Vashishtha *et al*. [5] For example, Vashishtha *et al*. [5] report European countries based on northern, southern, western and eastern Europe. However, because of the low number of harm trend records returned for this region, we have combined all records and reported them together as ‘mainland Europe.’ The implications for this are discussed in the limitations section. Other approaches to presenting the results were attempted, with the results of this found in Table S8. However, none of the approaches were sufficient in making the harm trends clearer to understand. Therefore, the results are presented geographically.

Results are presented via narrative synthesis according to the synthesis without meta‐analysis (SWiM) reporting guidelines, which can be found in Table S9 [88]. A statistical data synthesis was not completed largely because of the heterogeneity of the data—our results include a variety of time periods, measurements of harm and methods of measuring said harms. Unfortunately, this precludes us from conducting a meta‐analysis. Additionally, the aim of our review is to investigate country and region‐specific trends, rather than over‐arching global trends. With this in mind, our results are outlined below in text and tables according to the three geographic regions outlined above.

### Trends in alcohol‐related harm

#### North America

There were 11 records from North America, with seven (64%) showing either decreasing or stable trends, one (9%) showing increasing trends and three (27%) showing mixed results because of sex‐based differences (Table 6). Results from the four records with shorter timespans can be found in Table S10.

**TABLE 6:** Key results regarding trends in alcohol‐related harms for records over a 5‐year timespan (*n* = 37 total)

Country	First author, publication year OR data summary	Direction of trend	Proportion	Timespan	Measure
**North America (*n* = 11 records)**					
US	Moise, 2019 [62]	Decrease	56% decline in rates for 10‐ to 14‐year‐olds 36% decline in rates for 15‐ to 19‐year‐olds	Comparing 2006–2010 to 2011–2015	Age‐standardised rates of alcohol‐positive unintentional injury hospitalisations
US	White, 2018 [64]	Decrease	APC of −2.7 for 12‐ to 17‐year‐olds	2006–2014	Rates of all alcohol‐related ED visits per 100 000 population
US	On‐line report titled: Trends in alcohol‐related morbidity among community hospital discharges [65]	Decrease	39% decrease in rates for 12‐ to 20‐year‐olds	2005–2015	Principal alcohol‐related discharges for 12‐ to 20‐year‐olds per 10 000 population
Canada^a^	On‐line database titled: Canadian substance use costs and harms [60]	Decrease	59% decrease in rates for 0‐ to 14‐year‐olds 28% decrease for 0‐ to 14‐year‐old males 70% decrease for 0‐ to 14‐year‐old females	2007–2019	Unstandardized rates of alcohol‐attributable ED visits per 100 000
Canada^a,^	On‐line database titled: Canadian substance use costs and harms [60]	Stable	Stability in rates for 0‐ to 14‐year‐olds	2007–2019	Unstandardized rates of alcohol‐attributable hospitalisations per 100 000.
US	Downloadable dataset, page titled: National estimates of drug‐related emergency department visits, 2004–2011 – All visits [67]	Stable	Stability in rates for all age groups (12‐ to 17‐year‐olds, 18‐ to 20‐year‐olds and under 21‐year‐olds)	2005–2011	Estimated rates of alcohol‐related ED visits per 100 000 population
US	Ngo, 2018 [61]	Increase	63% increase in rates for under 20‐year‐olds	2009/2010–2014/2015	Rates of alcohol‐related ED presentations per 100 ED visits
Canada^a^	Smith, 2023 [59]	Mixed	43% increase in the rates for 15‐ to 34‐year‐old females Stability in rates for 15‐ to 34‐year‐old males	2008–2019	Rates of alcohol‐attributable hospitalisations per 100 000 population
Canada^a^	Smith, 2023 [59]	Mixed	17% increase in rates for 15‐ to 34‐year‐old females 16% decrease in rates for 15‐ to 34‐year‐old males	2008–2019	Rates of alcohol‐attributable ED visits per 100 000 population
Canada	Myran, 2019 [76]	Mixed	21% increase in rates for 10‐ to 18‐year‐old females Stability in rates for 10‐ to 18‐year‐old males	2005–2016	Rates of alcohol‐related ED visits per 100 000 population
North America^b^	Danpanichkul *et al*., 2024 [29]	Mixed	Stability in APC for Canada Decrease in APC (−1.85%) for US (*P* < 0.001)	2000–2019	APC for alcohol‐related cases of chronic liver disease and cirrhosis
**Other Anglosphere countries (*n* = 13 records)**					
UK: Wales	Trefan, 2019 [71]	Decrease	38% less likelihood of hospital admission for 10‐ to 17‐year‐olds (*P* < 0.001)	Comparing 2006 to 2011	Rates of alcohol‐related hospital admissions per 1000 population
UK: England	Green, 2017 [73]	Decrease	N/A – No raw data provided.	2005/2006–2013/2014	Rates of acute hospital admissions wholly attributable to alcohol per 100 000 population
UK^c^: England	O'Donnell, 2017 [58]	Decrease	29% decrease in rates for 13‐ to 17‐year‐olds. From 2005, admissions declined by 6% each year (*P* < 0.01).	1997–2012	Rates of alcohol‐related admissions per 10 000 population
Australia	Sims, 2021 [78]	Decrease	Annual percentage change (APC) of −6.2 for 12‐ to 14‐year‐olds (−5.8 for males and −6.6 for females) APC of −4.2 for 15‐ to 17‐year‐olds. (−4.6 for males and −3.7 for females)	2005/2006–2016/2017	Rates of alcohol‐related ED presentations (after matching with subsequent hospitalisations) per 10 000 population
New Zealand	Downloadable dataset, page titled: Alcohol‐related harm data [85]	Decrease	73% decrease in rates for 0‐ to 14‐year‐olds 11% decrease in rates for 15‐ to 24‐year‐olds	2005–2019	Rates of hospitalisations wholly attributable to alcohol per 100 000
UK: England	On‐line database titled: Local Alcohol Profiles for England [68]	Decrease	56% decrease in rates for all under 18‐year‐olds 58% decrease for males, 55% decrease for females	2006/2007–2018/2019	Rates of hospital admissions wholly attributable to alcohol per 100 000 population
UK: Scotland	Downloadable dataset, page titled: Alcohol‐related hospital statistics [69]	Decrease	55% decrease in rates for all under 15‐year‐olds 57% decrease for males, 54% decrease for females 30% decrease in rates for 15‐ to 19‐year‐olds 32% decrease for males, 27% decrease for females	2005/2006–2018/2019	Rates of alcohol‐related hospital admissions per 100 000 population
Ireland	Online report titled: Alcohol consumption, alcohol‐related harm and alcohol policy in Ireland [83]	Decrease	90% decrease in proportion of 0‐ to 17‐year‐olds contributing to all alcohol‐related discharges 73% decrease for both 0‐ to 17‐year‐old males and females	Comparing 1995 to 2018	Proportion of the 0‐ to 17‐year‐old age group making up the total amount of hospital discharges wholly attributable to alcohol
Australia	Mitra, 2023 [77]	Stable	5% less likelihood trauma centre presentation for 16‐ to 19‐year‐olds	2008–2019	Rates of alcohol‐positive presentations to an ED trauma centre
UK: England	Tyrrell, 2018 [70]	Mixed	67% decrease in rates for 10‐ to 14‐year‐old males Unclear, but likely stable, for 10‐ to 14‐year‐old females	1998–2014	Rates of alcohol‐related poisoning events per 100 000 population
Australia	On‐line database titled: Australian alcohol‐attributable harm visualisation tool [79]	Mixed	Stability in rates for 15‐ to 34‐year‐olds 14% decrease in rates for 15‐ to 34‐year‐old males Stability in rates for 15‐ to 34‐year‐old females	2011–2019	Age‐specific rates of alcohol‐attributable hospitalisations per 100 000 population
Australia	Downloadable dataset, page titled: Alcohol‐related injury: hospitalisations and deaths, 2019–2020 [80]	Mixed	21% decrease in numbers for 15‐ to 24‐year‐olds 29% decrease in numbers for 15‐ to 24‐year‐old males Stability in numbers for 15‐ to 24‐year‐old females	2010/2011–2016/2017	No. of alcohol‐related injury hospitalisations
Other Anglosphere countries^b^	Danpanichkul *et al*., 2024 [29]	Mixed	Stability in APC for Australia, New Zealand and the United Kingdom Increase in APC (0.73%) for Ireland (*P* < 0.001)	2000–2019	APC for alcohol‐related cases of chronic liver disease and cirrhosis
**Mainland Europe (*n* = 13 records)**					
Sweden	On‐line database titled: Statistical database, diagnoses [86]	Decrease	32% decrease in rates for 10‐ to 19‐year‐olds 33% decrease in rates for 10‐ to 19‐year‐old males 30% decrease in rates for 10‐ to 19‐year‐old females	2008–2019	Age‐standardised rates of alcohol‐related in‐patient or specialised open care (ED or hospital admissions) per 100 000 population
Spain	On‐line report titled: Mortality Attributable to Alcohol in Spain (Mortalidad Atribuible al Alcohol en España) [53]	Decrease	70% decrease in rates for 15‐ to 34‐year‐old excessive drinkers 56% decrease in rates for 15‐ to 34‐year‐old light/moderate drinkers.	Comparing 2001–2009 to 2010–2017	Mortality rates attributable to alcohol per 100 000 population
Austria	Downloadable dataset, page titled: Hospital Discharges from Acute Hospitals (Spitalsentlassungen aus Krankenanstalten) [54]	Decrease	48% decrease in numbers for 0‐ to 14‐year‐olds 51% decrease in numbers for 0‐ to 14‐year‐old males 46% decrease in numbers for 0‐ to 14‐year‐old females	2005–2019	No. of alcohol‐related hospital discharges
Sweden	Stafström, 2023 [18]	Decrease	Annual decrease of −7.15 (*P* < 0.001) for 15‐ to 19‐year‐old males Annual decrease of −7.75 (*P* < 0.001) for 15‐ to 19‐year‐old females	2000–2021	Linear regression of alcohol‐related harm indicators per 10 000 population
Estonia	On‐line report titled: Alcohol Market, Consumption and Harms in Estonia – Yearbook (Alkoholi turg, tarbimine ja kahjud Eestis – Aastaraamat) [32, 33, 34, 35, 36, 37]	Stable	Stability in numbers of patients age 16‐ to 20‐year‐olds	2013–2019	No. of patients turning to specialists or family doctors because of alcohol
Lithuania	On‐line database titled: Prevalence – number of ill people [57]	Stable	Stability in rates for 10‐ to 19‐year‐olds	2014–2019	Rates of alcohol‐related diagnoses in healthcare institutions and on death certificates, per 1000
Belgium	Panken, 2022 [81]^d^	Increase	Increase in numbers for 10‐ to 19‐year‐olds (but no raw data provided)	2008–2019	No. of alcohol‐positive ED admissions for entire population in a student city
The Netherlands	On‐line database titled: Hospital admissions and patients (Ziekenhuisopnamen en ‐patiënten) [55]	Increase	21% increase in rates for 1‐ to 20‐year‐olds 12% increase in rates for 1‐ to 20‐year‐old males 22% increase in rates for 1‐ to 20‐year‐old females	2013–2019	Rates of alcohol‐related hospital admissions per 10 000 inhabitants
Switzerland	On‐line database titled: Patients in hospitals by age class, sex and diagnostic group (Patients dans les hôpitaux selon la classe l'âge, le sexe et le groupe de diagnostic) [56]	Increase	94% increase in numbers for 10‐ to 14‐year‐olds 30% increase in numbers for 15‐ to 19‐year‐olds	2005–2019	No. of alcohol‐related hospital admissions
Germany	On‐line database titled: Diagnostic data of the hospitals starting from 2000 [87]	Mixed	17% decrease in rates for 10‐ to 14‐year‐old males 31% increase in rates for 10‐ to 14‐year‐old females 19% increase in rates for 15‐ to 19‐year‐old males 47% increase in rates for 15‐ to 19‐year‐old females	2005–2019	Rates of hospital discharges for alcohol‐related diagnoses per 100 000 inhabitants
Switzerland	Wicki, 2020 [84]	Mixed	31% decrease in rates for 10‐ to 15‐year‐olds Stability in rates for 16‐ to 19‐year‐olds	2010–2016	Proportion rates of alcohol‐related hospitalisations when accounting for Switzerland and excluding the Canton of Vaud (area of alcohol policy changes)
Finland	On‐line report titled: Yearbook of Alcohol and Drug Statistics (Päihdetilastollinen vuosikirja; Alkoholi ja huumeet) [38, 39, 40, 41, 42, 43, 44, 45, 46, 47, 48, 49, 50, 51, 52]	Mixed	Stability in numbers for 0‐ to 14‐year‐olds 30% decrease in numbers for 15‐ to 19‐year‐olds	2005–2019	No. of hospital inpatient care periods with a primary diagnosis of alcohol
Mainland Europe^b^	Danpanichkul *et al*., 2024 [29]	Mixed	Stability in APC for Finland, Germany and the Netherlands Increases in APC for Belgium (0.83%), Estonia (1.26%), Iceland (0.92%), Lithuania (1.54%), Norway (0.77%) and Switzerland (1.19%). *P* < 0.001 Decreases for Austria (−0.86%), Portugal (−1.93%), Spain (−0.72%), Sweden (−0.57%). *P* < 0.001	2000–2019	APC for alcohol‐related cases of chronic liver disease and cirrhosis

Abbreviations: APC, annual percentage change; ED, emergency department; UK, United Kingdom; US, United States.

^a^ Source has been duplicated into two records to reflect differing results for separate hospital admissions and ED results.

^b^ Source has been split into three records to reflect differing results for geographic regions. The original source includes results for each individual country included in this study—see Table S3 for further detail.

^c^ Source has been split into two records to reflect differing results for separate countries (Australia and the UK).

^d^ Indicates records that scored a moderate risk of bias according to Hoy *et al*. [28].

Of the records that indicated a decrease in rates of alcohol‐related harm over time, two focused on ED visits and three on hospitalisation data. Only one record from this region reported increases in rates of alcohol‐related harm [61]. This record consists of data from a single college‐specific ED in the United States, with the results presented as the proportion of all ED visits, rather than overall rates of alcohol‐related ED visits over time. When considering records detailing sex‐specific trends (*n* = 4), all of which report on data from Canada, the results are conflicting. For females, three of the four records indicated an increase of up to 43% in the rates of alcohol‐related harms [59, 76], whereas another reported a decrease of 70% [60]. Comparatively, records for males indicated either decreasing (in three records [59, 60, 76]) or stable results (in one record [59]).

When combining consumption trends from both the United States and Canada, Vashishtha *et al*. [5] reported an average 39% decline in past‐month drinking. Comparatively, our harm records either closely mirrored consumption trends or varied depending on the age group studied, with harm decreases of 56% reported for 10‐ to 14‐year‐olds, but decreases of only 36% for 15‐ to 29‐year‐olds [62]. Our records from Canada, where Vashishtha *et al*. [5] reported a 38.4% decrease in consumption, indicated conflicting sex‐based differences in harm trends—with three records noting increases in harms for females against a backdrop of stability or decreases for males [59], whereas another noted decreases for both sexes.

Overall, results from North America indicated mostly decreasing trends in alcohol‐related harms, with some rates remaining relatively stable. Notably, there is some, albeit conflicting, evidence to suggest an increase in alcohol‐related harms for females, whereas results for males consistently indicated decreases or stabilisation throughout multiple time periods and age groups.

#### Other Anglosphere countries

Thirteen records were included from other Anglosphere countries. Of these, eight (61%) reported decreasing trends, one (8%) reported stability and four (31%) reported mixed trends, mostly because of sex‐specific differences. Notably, there was only a single record from this region that indicated an increase in rates of harms, which was for Ireland [29], despite the only other source from this country indicating decreases in harms [83]. The key results can be found in Table 6, with results from the two records with shorter timespans found in Table S10.

The majority (58%; *n* = 7) of the records indicating decreases in alcohol‐related harms used hospital admissions data, with a single record using ED data. Many records reported sizeable decreases in harms. Records reporting stable trends for New Zealand, the United Kingdom and Australia included hospitalisations [29] and for Australia, ED presentations as well [77]. This ED‐based study identified a significant decrease in harms, but the overall decline was only 5% so we have classified it as a stable trend based on our specified 10% threshold. Seven records investigated sex‐based differences. Although four of these indicated similarly decreasing rates of harm for both sexes [68, 69, 78, 83], three records noted mixed results, with decreasing rates for males contrasting with stable trends for females [70, 79, 80].

Vashishtha *et al*. [5] recorded an average 50% reduction in consumption across the four Anglosphere regions we collected data for—Australia, New Zealand, the United Kingdom and Ireland. The majority decreasing or stable rates of alcohol‐related harms noted above indicate that harm trends were broadly consistent with declines in consumption, although the magnitude of these declines occasionally varied. For example, in New Zealand, Vashishtha *et al*. [5] reported a 43% reduction in drinking. Our record for this country indicated a 73% decrease in harms for those ages 0 to 14, but only an 11% decrease for 15‐ to 24‐year‐olds [85].

Overall, the vast majority of results from this region indicated decreasing or stable results, with decreasing being the most reported trend direction. In general, decreases in alcohol‐related harms were noted more in male populations, whereas for some records, female populations were more likely to report stable results.

#### Mainland Europe

A total of 13 records were located from mainland Europe, with four records (31%) indicating decreases in alcohol‐related harms, three (23%) increases, two (15%) stability and four (31%) mixed results (Table 6). Records with shorter timespans can be found in Table S10.

Decreases in alcohol‐related harms were found in three records for Sweden [18, 29, 86], two for Spain [29, 53] and for Austria [29, 54] and in one record for Portugal [29]. Measures of harm varied and most records indicated sizeable decreases—for example, a 70% decrease in alcohol‐related mortality for excessive drinkers age 15 to 34 in Spain [53]. Results from Lithuania [57], Estonia [32, 33, 34, 35, 36, 37], Finland, Germany and the Netherlands [29] reported stability over time.

Results from Belgium, the Netherlands and Switzerland indicated increases in alcohol‐related harms [55, 56, 81]. However, it should be noted that the data from Belgium focuses on ED admissions in a student city, and while they note that those age 10 to 19 remained a stable proportion of the overall increasing numbers of admissions [81], there was no data provided about this specific age group. Results from Danpanichkul *et al*. [2] also reported increases for Belgium and Switzerland, as well as Estonia, Iceland, Lithuania and Norway.

Three records from Germany, Switzerland and Finland indicated mixed results. The record from Germany indicated increases in rates of alcohol‐related hospital discharges for all groups except 10‐ to 14‐year‐old males, where a 17% decrease was reported [87]. The record from Switzerland noted a decrease in harm rates for younger (10–15) adolescents, but stability in rates for older (16–19) adolescents [84], whereas the record from Finland noted the reverse: stability for younger (0–14) adolescents, and decreases for older (15–19) adolescents [38, 39, 40, 41, 42, 43, 44, 45, 46, 47, 48, 49, 50, 51, 52].

The four records detailing sex‐specific trends were largely uniform. Where decreases or increases in harms were noted, these harms broadly declined or increased at similar levels for both males and females [54, 55, 86]. The only exception to this was the aforementioned record from Germany [87], where younger males decreased whereas older males and all female age groups increased.

The reductions in past‐month consumption ranged between 33% and 61% in mainland Europe [5], which Vashishtha *et al*. grouped as northern, southern, western and eastern Europe. Across our records, the decreases in harms for northern and southern Europe followed these declining consumption trends, albeit at varying magnitudes, with some records indicating larger decreases in harms than consumption. For example, in Spain a 35.7% reduction in consumption was reported [5], whereas harm trends indicated a 70% decrease in mortality rates for excessive drinkers [53]. Our results for western and eastern Europe were more mixed, including increases in harm reported particularly for western Europe (e.g. the Netherlands [55]). Contradictory evidence was also found within countries—where Vashishtha *et al*. [5] reported a 44.9% decrease in consumption in Switzerland, we found one record noting a 31% decrease per 1000 hospital admissions for 10‐ to 15‐year‐olds [84], whereas another indicated a 94% increase in the numbers of hospital admissions for roughly the same age group (10‐ to 14‐year‐olds) [56].

Overall, results from Europe were contradictory. Although this was the region with the most increases reported, there does not seem to be one clear trend overall, with some countries even indicating different trend directions across multiple records (e.g. Switzerland). It should be noted that many records in this region had methodological or reporting issues (e.g. consistency issues across the yearly reports for Estonia [32, 33, 34, 35, 36, 37]), which may make the results less reliable.

## DISCUSSION

This review is the first to examine trends in adolescent alcohol‐related harms in countries where consumption has declined. The results indicate that harms have decreased simultaneously with consumption in many countries. This evidence was strongest in the United Kingdom, Australia, New Zealand and Ireland. Harm trends in North America followed consumption to a lesser degree, with evidence of increases in harms for college populations and mixed evidence for increases in harms for females in Canada. The low number of results from Europe indicated contradictory results with no clear trend, with decreasing harm trends inconsistently matching declining consumption trends.

It is possible that the unclear results from Europe were because of a lack of data for this region. Three countries in this region were represented by a single record, with a further six represented only by two records—compared to the North America region where we had 10 records for the United States and six for Canada, strengthening the consistency of these results. Our study highlights the need for more data to be made publicly available in the European region, however, it is also possible that our limited findings were because of our English‐language search restriction.

The results indicate some differences between trends in alcohol‐related harms and trends in consumption, with declines in consumption generally more dramatic than those for harms. However, it should be noted that our measure of consumption was self‐reported prevalence of past‐month drinking, whereas our measures of harm were usually annual harm rates. Another reason for this discrepancy is that there are distinct populations contributing to consumption and harm data. Research indicates that survey sampling (such as that used in Vashishtha *et al*.’s [5] consumption data) tend to miss key subpopulations, such as non‐responders—who are more likely to report heavier alcohol use [89]. Heavier alcohol use is associated with increased rates of experiencing alcohol‐related harms [90, 91]. Therefore, it is possible that the adolescents who are experiencing the alcohol‐related harms reflected in our data have mostly been missed in the surveys used to compile consumption trend data. Further reasons for the differences in harm and consumption trends may be because of limitations in our study design and are discussed below.

Despite the differences in magnitudes of rates, our results indicate that for many countries, adolescent alcohol‐related harms are decreasing over time, mirroring the well‐researched trends of declining adolescent consumption [5]. These results are indicative of a potential decrease in the substantial proportion of disease burden attributable to alcohol [2]. Further work is required to understand the seemingly contradictory results indicating increases in harms for some female and college/university populations amidst a backdrop of broadly declining consumption rates. Additionally, emerging qualitative work suggests that alcohol consumption in university populations may not have declined as much as the general younger population, which could explain the discrepancies in harm rates for these groups [92]. Given that declines in the prevalence of consumption are also present for young adults in some high‐income countries [23], further work replicating our search strategies for this young adult population would be valuable in understanding if declines in alcohol‐related harms are also seen for this age group.

### Limitations

There are some key methodological issues to note. The primary outcome was the percentage change in alcohol‐related harms between the start and end of each study's time period, with a 10% threshold applied. This was a pragmatic choice. Given much of the included data covered varying time frames and reported only descriptive results (i.e., with no statistical testing), the authors decided on a 10% threshold to avoid over‐interpreting minor changes in population rates as meaningful. We explored the implications of this choice using sensitivity analyses. As well, studies with shorter timespans were moved to the supplementary materials. Additionally, although a systematic approach was adhered to where possible, the grey literature search does include more variability, which potentially introduced bias into this study. For example, the authors relied partly on contact experts to locate data for specific countries—however, this method has been supported in previous research [27] and was an essential component of the grey literature search.

The current results are also largely unable to account for changes in countries' reporting practices over time. For example, data from Sweden note that reporting practices have improved over the years and that large changes in prevalence rates may be because of improved reporting and not an increase in adolescents seeking medical care [86]. These concerns are mirrored in some Australian data, where data quality can depend on the coding practices of hospital staff, as it may not be mandatory for EDs to collect information [78, 80]. Additionally, our grey literature search potentially missed key data as we were unable to include reports not provided in English, and because of our lack of key research contacts for such countries.

Overall, the results of this review are largely heterogeneous—with differences in harm outcomes (e.g. comparing hospitalisations and mortality data), measurement types (e.g. comparing standardised rates and raw numbers), age groups and time periods. It is possible that this in itself explains why such a variety of trend directions were found across countries. Because of this heterogeneity, there were also difficulties in presenting the results, as previously noted in the methods section (see Table S8). Although we chose to present the results geographically, this method is not without its limitations. Our results report on mainland Europe as a single region because of low record numbers for this area, however, Vashishtha *et al*. [5] reported differences in adolescent consumption trends for specific sections of Europe. It is, therefore, unsurprising that this region reported the most mixed harm trends. This further reinforces the need for more publicly accessible alcohol‐related harm data to be made available for this region, as there seems to be for consumption trends [5].

## CONCLUSION

The current study was the first to assess trends in adolescent alcohol‐related harms, with findings suggesting that harms have mostly declined in line with consumption, particularly in Anglosphere countries, although trends in North America and mainland Europe were more mixed. Where declines were not present, a stabilisation in harm trends was usually noted. Although there were some subpopulations where harms increased and relatively limited data was available for Europe, our findings demonstrate that since the decline in adolescent consumption from the mid‐2000s, alcohol‐related harms have similarly been declining in many countries. Future research could explore if this decline in harms extends to young adults and why some subpopulations showed increasing harm trends.

## AUTHOR CONTRIBUTIONS

**Emma Vieira:** Formal analysis (equal); investigation (lead); methodology (supporting); writing—original draft (lead); writing—review and editing (equal). **Nic Taylor:** Formal analysis (equal); investigation (supporting); methodology (supporting); supervision (supporting); writing—original draft (supporting); writing—review and editing (equal). **Abigail Stevely:** Investigation (supporting); writing—review and editing (equal). **Amy Pennay:** Investigation (supporting); writing—review and editing (equal). **Jonas Raninen:** Investigation (supporting); writing—review and editing (equal). **John Holmes:** Investigation (supporting); writing—review and editing (equal). **Rakhi Vashishtha:** Investigation (supporting); writing—review and editing (equal). **Michael Livingston:** Conceptualization (lead); formal analysis (equal); investigation (supporting); methodology (lead); supervision (lead); writing—original draft (supporting); writing—review and editing (equal).

## DECLARATION OF INTERESTS

None.

## Supporting information


**Table S1.** Table indicating trends in adolescent past‐month consumption from final year studied in Vashishtha et al. [5] until 2019 for n = 19 countries included in review.
**Material S1.** Detailed search strategy, screening and data extraction process.
**Table S2.** Search strings and results for systematic review search.
**Table S3.** Summary of results from Danpanichkul et al. [29] indicating annual percentage change from 2000 to 2019 in rates of alcohol‐associated liver disease for 15–19‐year‐olds by country.
**Table S4.** Summary of data extraction for n = 9 records with time spans of 5 years or less in the period of interest (2005 onward).
**Table S5.** Specific harm measurements for n = 41 sources.
**Table S6.** Quality assessment results table for n = 41 sources.
**Table S7.** Instances where source variables are included despite being outside the original eligibility criteria and other biases or limitations noted within included studies.
**Table S8.** Alternative approaches to presenting harm trend results for n = 37 records with timespans over 5 years.
**Table S9.** Table reporting synthesis without meta‐analysis (SWiM) reporting guidelines.
**Table S10.** Key results regarding trends in alcohol‐related harms for n = 9 records with time spans of 5 years or less in the period of interest.

## Data Availability

Data sharing is not applicable to this article as no new data were created or analysed in this study.
