# Supplementary material for: A systematic review of adolescent alcohol‐related harm trends in high‐income countries with declines in adolescent consumption
Source: Addiction. 2025 Mar 5;120(8):1551–70. doi: 10.1111/add.70026 (PMC12215224; doi:10.1111/add.70026)
Supplement: Supplementary file 1 — Table S1. Table indicating trends in adolescent past‐month consumption from final year studied in Vashishtha et al. [5] until 2019 for n = 19 countries included in review. Material S1. Detailed search strategy, screening and data extraction process. Table S2. Search strings and results for systematic review search. Table S3. Summary of results from Danpanichkul et al. [29] indicating annual percentage change from 2000 to 2019 in rates of alcohol‐associated liver disease for 15–19‐year‐olds by country. Table S4. Summary of data extraction for n = 9 records with time spans of 5 years or less in the period of interest (2005 onward). Table S5. Specific harm measurements for n = 41 sources. Table S6. Quality assessment results table for n = 41 sources. Table S7. Instances where source variables are included despite being outside the original eligibility criteria and other biases or limitations noted within included studies. Table S8. Alternative approaches to presenting harm trend results for n = 37 records with timespans over 5 years. Table S9. Table reporting synthesis without meta‐analysis (SWiM) reporting guidelines. Table S10. Key results regarding trends in alcohol‐related harms for n = 9 records with time spans of 5 years or less in the period of interest. [file ADD-120-1551-s001.docx]

**Supplementary Materials – Contents**

[**Supplementary Table 1.** Table indicating trends in adolescent past-month consumption from final year studied in Vashishtha et al. (5) until 2019 for n = 19 countries included in review. 2](#_Toc187252443)

[**Supplementary Material 1.** Detailed search strategy, screening and data extraction process. 4](#_Toc187252444)

[**Supplementary Table 2.** Search strings and results for systematic review search. 6](#_Toc187252445)

[**Supplementary Table 3.** Summary of results from Danpanichkul et al. (29) indicating annual percentage change from 2000-2019 in rates of alcohol-associated liver disease for 15-19-year-olds by country. 9](#_Toc187252446)

[**Supplementary Table 4.** Summary of data extraction for n = 9 records with time spans of 5 years or less in the period of interest (2005 onwards). 10](#_Toc187252447)

[**Supplementary Table 5.** Specific harm measurements for n = 41 sources. 11](#_Toc187252448)

[**Supplementary Table 6.** Quality assessment results table for n = 41 sources. 16](#_Toc187252449)

[**Supplementary Table 7.** Instances where source variables are included despite being outside the original eligibility criteria and other biases or limitations noted within included studies. 18](#_Toc187252450)

[**Supplementary Table 8.** Alternative approaches to presenting harm trend results for n = 37 records with timespans over 5 years. 20](#_Toc187252451)

[**Supplementary Table 9.** Table reporting synthesis without meta-analysis (SWiM) reporting guidelines. 21](#_Toc187252452)

[**Supplementary Table 10.** Key results regarding trends in alcohol-related harms for n = 9 records with time spans of 5 years or less in the period of interest. 23](#_Toc187252453)

## **Supplementary Table 1.** Table indicating trends in adolescent past-month consumption from final year studied in Vashishtha et al. (5) until 2019 for n = 19 countries included in review.

| **Country** | **End year by Vashishtha et al. 2020** | **Trend until 2019** | **Details of trend** | **Years** | **Source** | **Age group** |
| --- | --- | --- | --- | --- | --- | --- |
| Australia | 2017 | Decline | 26.5%-22.2% | 2017-2022/23^a^ | Australian Secondary Students’ Alcohol and other Drug Survey(82) | 12-17 years |
| Austria | 2013/14 | Decline | 68-63% | 2015-2019 | European School Survey Project on Alcohol and Other Drugs (ESPAD) Survey(20) | 15-16 years |
| Belgium | 2013/14 | Decline | Girls: 15.5-13%  Boys: 20.5-15%  (Average across Flemish & French) | 2014-2018 | Health Behaviour in School-aged Children (HBSC) Survey(79) | 13 years |
| Canada | 2013/14 | Stable | Girls: 12%  Boys: 12% | 2014-2018 | HBSC Survey(79) | 13 years |
| Estonia | 2015 | Decline | 38-37% | 2015-2019 | ESPAD Survey(20) | 15-16 years |
| Finland | 2015 | Decline | 32-30% | 2015-2019 | ESPAD Survey(20) | 15-16 years |
| Germany | 2013/14 | Decline | 67-65% | 2015-2019 | ESPAD Survey(20) | 15-16 years |
| Iceland | 2015 | Increase | 9-11% | 2015-2019 | ESPAD Survey(20) | 15-16 years |
| Ireland | 2013/14 | Increase | 35-41% | 2015-2019 | ESPAD Survey(20) | 15-16 years |
| Lithuania | 2015 | Decline | 34-27% | 2015-2019 | ESPAD Survey(20) | 15-16 years |
| The Netherlands | 2013/14 | Decline | Girls: 11-7%  Boys: 11-10% | 2014-2018 | HBSC Survey(79) | 13 years |
| New Zealand | 2012 | Decline | 36-34% | 2012-2019 | Youth 2000 Health Survey(80) | Approx. 13-17 years |
| Norway | 2015 | Increase | 22-25% | 2015-2019 | ESPAD Survey(20) | 15-16 years |
| Portugal | 2015 | Increase | 42-43% | 2015-2019 | ESPAD Survey(20) | 15-16 years |
| Spain | 2013/14 | Increase | Girls: 10-11%  Boys: 9-10% | 2014-2018 | HBSC Survey(79) | 13 years |
| Sweden | 2015 | Decline | 26-25% | 2015-2019 | ESPAD Survey(20) | 15-16 years |
| Switzerland | 2013/14 | Stable | Girls: 10%  Boys: 14% | 2014-2018 | HBSC Survey(79) | 13 years |
| The United Kingdom: England | 2013/14 | Increase | Girls: 15-23%  Boys: 15-19% | 2014-2018 | HBSC Survey(79) | 13 years |
| The United Kingdom: Scotland | 2013/14 | Increase | Girls: 13-14%  Boys: 11-13% | 2014-2018 | HBSC Survey(79) | 13 years |
| The United Kingdom: Wales | 2013/14 | Increase | Girls: 19-24%  Boys: 19-24% | 2014-2018 | HBSC Survey(79) | 13 years |
| The United Kingdom: Northern Ireland | 2013/14 | Decline | 22-16% | 2013-2019 | Young Persons’ Behaviour and Attitudes Survey(83, 84) | 11-16 years |
| The United States of America | 2017 | Decline | 29.8-29.2% | 2017-2019 | Youth Risk Behaviour Survey(81) | Approx. 14-18 years |

*Note: All sources reflect those in Vashishtha et al.’s (2020) original analysis, using past-month measures of adolescent alcohol consumption.*

*^a^Due to Covid-19, the 2019 survey was not completed and the most recent survey is the 2022/23 survey.*

## **Supplementary Material 1.** Detailed search strategy, screening and data extraction process.

**Systematic search strategy**

The databases Medline, CINAHL, Scopus and PubMed were systematically searched by EV on 30/05/2023. Two additional searches were conducted on 19/06/2024 and 07/01/2025 to identify any further studies published since the initial search. These searches resulted in the inclusion of four additional studies(18, 29-31). A further source, comprising of several yearbook reports for Finland, was also added after peer-review suggestion (35-49). The terms used in the searches included the population of adolescents, alcohol-related terms, health-system based measurements of harm, and trend-related terms. The complete list of search terms can be seen in the table below. Only titles and abstracts were searched, and the search was limited to sources in English published from 2005 onwards. The entire systematic search strings and results can be found in Supplementary Table 2.

**Table indicating complete list of search terms used for systematic search.**

| **Adolescent-related terms** | **Alcohol-related terms** | **Harm-related terms** | **Trend-related terms** |
| --- | --- | --- | --- |
| Youth* | Alcohol* | Emergency | Trend* |
| Adolescen* | Drink* | Hospital? | Declin* |
| Under-age | Binge-drinking | Presentation* | Inclin* |
| Young people | Intoxication | Admission* | Longitudinal |
| Young person? | Under-age drinking | Accident* | Gradual |
| Young adult? |  | Mortality | Slope |
| Juvenile? |  | Death* |  |
| Minor? |  | Injur* |  |
| Student* |  | Alcohol-related harm* |  |
| Teen* |  | Morbidit* |  |
|  |  | Ambulance* |  |
|  |  | Paramedic* |  |
|  |  | Harm* |  |

**Grey literature search strategy**

Much of the data regarding alcohol-related harms is recorded in government monitoring and reporting systems. Therefore, a search of the grey literature was essential for this review, although the methods for this are less replicable. We focused our grey literature searches on data that was already compiled and accessible to the general public via Google searches, rather than contacting governments or other agencies to apply for bespoke access to data.

Following the method outlined by Godin et al.(27), EV, NT and ML searched for and screened the grey literature by checking the first ten pages of Google with one of the following search strings: [country] alcohol harms, [country] adolescent alcohol harms, [country] alcohol statistics, or [country] alcohol harm rates. Each country was randomly assigned to one of the above three searchers, and was then checked by another to ensure no sources were missed. Additionally, experts from eight countries were contacted by ML to assist in identifying grey literature for that country(27) - see Table 2 for the list of countries and results for this method. Finally, backwards snowballing was utilised on all the peer-reviewed studies that progressed to the full-text screening stage (n = 91). Table 2 indicates the total number of grey literature sources that were identified from the various methods outlined above, which included online databases, downloadable datasets, and online reports.

**Systematic searches screening and data extraction**

As the authors only had access to the ‘free trial’ version of Covidence, title and abstract screening were conducted in Endnote by one reviewer (EV). A secondary reviewer (NT) was randomly assigned a 10% subset of all records to check the screening, with full agreement between the two reviewers found. The remaining 114 studies eligible for full-text screening were imported into Covidence for the remaining screening and data extraction (if eligible).

Data was extracted from the 18 sources using a standardised form developed a-priori by the research team (see Table 3). Extraction was completed in Covidence by EV (56%; n = 10), NT (22%; n = 4), and ML (22%; n = 4), with a randomly assigned secondary researcher checking and confirming each data extraction. The secondary checkers were not blind to the original data extraction, as their role was primarily to confirm the initial data extraction and highlight any discrepancies. Any disagreements between reviewers during extraction were decided by the senior author (ML).

**Grey literature searches data extraction**

Screening and data extraction for the grey literature were completed simultaneously, as the nature of the types of sources included in the grey literature search precluded them from being imported into Endnote or Covidence (i.e., Covidence is useful for screening peer-reviewed journal articles, but the software is unable to import an international database). Therefore, once a source was identified via Google searches, it moved directly to the extraction phase. This was completed in Microsoft Excel using the same standardised form as the systematic searches (see Table 3). The researcher who initially identified the grey literature source was responsible for data extraction, with extraction completed by EV (48%; n = 11), ML (48%; n = 11) and NT (4%; n = 1). This extraction was then checked and confirmed by either EV, ML, or NT through random assignment, who were not blind to the initial data extraction (as per the systematic search data extractions).

## **Supplementary Table 2.** Search strings and results for systematic review search.

| Database | Search String | String Number | Number of Results |
| --- | --- | --- | --- |
| Medline | (alcohol* or drink* or binge-drinking or intoxication or under-age drinking).tw. | 1 | 531,224 |
|  | limit 1 to (english language and yr="2005 -Current") | 2 | 320,138 |
|  | (trend* or declin* or inclin* or longitudinal or gradual or slope).tw. | 3 | 1,326,378 |
|  | limit 3 to (english language and yr="2005 -Current") | 4 | 943,662 |
|  | (youth* or adolescen* or under-age or young people or young person? or young adult? or Juvenile? or minor? or student* or teen*).tw. | 5 | 1,180,003 |
|  | limit 5 to (english language and yr="2005 -Current") | 6 | 791,254 |
|  | (emergency or hospital? or presentation* or admission* or accident* or mortality or death* or injur* or alcohol-related harm* or harm* or morbidit* or ambulance* or paramedic*).tw. | 7 | 4,637,176 |
|  | limit 7 to (english language and yr="2005 -Current") | 8 | 3,109,697 |
|  | 2 and 4 and 6 and 8 | 9 | **974** |
| CINAHL | TI ( alcohol* or drink* or binge-drinking or intoxication or under-age drinking ) OR AB ( alcohol* or drink* or binge-drinking or intoxication or under-age drinking )  **Limiters** - Published Date: 20050101-; English Language | S1 | 112,471 |
|  | TI ( emergency or hospital? or presentation* or admission* or accident* or mortality or death* or injur* or alcohol-related harm* or harm* or morbidit* or ambulance* or paramedic* ) OR AB ( emergency or hospital? or presentation* or admission* or accident* or mortality or death* or injur* or alcohol-related harm* or harm* or morbidit* or ambulance* or paramedic* )  **Limiters** - Published Date: 20050101-; English Language | S2 | 1,102,787 |
|  | TI ( trend* or declin* or inclin* or longitudinal or gradual or slope ) OR AB ( trend* or declin* or inclin* or longitudinal or gradual or slope )  **Limiters** - Published Date: 20050101-; English Language | S3 | 298,479 |
|  | TI ( youth* or adolescen* or under-age or young people or young person? or young adult? or Juvenile? or minor? or student* or teen* ) OR AB ( youth* or adolescen* or under-age or young people or young person? or young adult? or Juvenile? or minor? or student* or teen* )  **Limiters** - Published Date: 20050101-; English Language | S4 | 421,456 |
|  | S1 AND S2 AND S3 AND S4 | S5 | **601** |
| Scopus | TITLE ( alcohol* OR drink* OR binge-drinking OR intoxication OR ( under-age AND drinking ) ) OR ABS ( alcohol* OR drink* OR binge-drinking OR intoxication OR ( under-age AND drinking ) ) AND PUBYEAR > 2004 AND ( LIMIT-TO ( LANGUAGE , "English" ) ) | 1 | 566,285 |
|  | TITLE ( emergency OR hospital? OR presentation* OR admission* OR accident* OR mortality OR death* OR injur* OR ( alcohol-related AND harm* ) OR harm* OR morbidit* OR ambulance* OR paramedic* ) OR ABS ( emergency OR hospital? OR presentation* OR admission* OR accident* OR mortality OR death* OR injur* OR ( alcohol-related AND harm* ) OR harm* OR morbidit* OR ambulance* OR paramedic* ) AND PUBYEAR > 2004 AND PUBYEAR < 2024 AND ( LIMIT-TO ( LANGUAGE , "English" ) ) | 2 | 4,146,506 |
|  | TITLE ( trend* OR declin* OR inclin* OR longitudinal OR gradual OR slope ) OR ABS ( trend* OR declin* OR inclin* OR longitudinal OR gradual OR slope ) AND PUBYEAR > 2004 AND PUBYEAR < 2024 AND ( LIMIT-TO ( LANGUAGE , "English" ) ) | 3 | 2,477,464 |
|  | TITLE ( youth* OR adolescen* OR under-age OR ( young AND people ) OR ( young AND person? ) OR ( young AND adult? ) OR juvenile? OR minor? OR student* OR teen* ) OR ABS ( youth* OR adolescen* OR under-age OR ( young AND people ) OR ( young AND person? ) OR ( young AND adult? ) OR juvenile? OR minor? OR student* OR teen* ) AND PUBYEAR > 2004 AND PUBYEAR < 2024 AND ( LIMIT-TO ( LANGUAGE , "English" ) ) | 4 | 1,733,923 |
|  | #1 AND #2 AND #3 AND #4 | 5 | **1060** |
| PubMed | alcohol*[Title/Abstract] OR drink*[Title/Abstract] OR binge-drinking[Title/Abstract] OR intoxication[Title/Abstract] OR under-age drinking[Title/Abstract]  Filters: English, from 2005 | 1 | 327,037 |
|  | emergency[Title/Abstract] OR hospital?[Title/Abstract] OR presentation*[Title/Abstract] OR admission*[Title/Abstract] OR accident*[Title/Abstract] OR mortality[Title/Abstract] OR death*[Title/Abstract] OR injur*[Title/Abstract] OR alcohol-related harm*[Title/Abstract] OR harm*[Title/Abstract] OR morbidit*[Title/Abstract] OR ambulance*[Title/Abstract] OR paramedic*[Title/Abstract]  Filters: English, from 2005 | 2 | 3,091,689 |
|  | trend*[Title/Abstract] OR declin*[Title/Abstract] OR inclin*[Title/Abstract] OR longitudinal[Title/Abstract] OR gradual[Title/Abstract] OR slope[Title/Abstract] Filters: English, from 2005 | 3 | 940,455 |
|  | youth*[Title/Abstract] OR adolescen*[Title/Abstract] OR under-age[Title/Abstract] OR young people[Title/Abstract] OR young person?[Title/Abstract] OR young adult?[Title/Abstract] OR Juvenile?[Title/Abstract] OR minor?[Title/Abstract] OR student*[Title/Abstract] OR teen*[Title/Abstract] Filters: English, from 2005 | 4 | 760,038 |
|  | 1 AND 2 AND 3 AND 4 | 5 | **931** |

Notes. Final result numbers are noted in bold.

## **Supplementary Table 3.** Summary of results from Danpanichkul et al.(29) indicating annual percentage change from 2000-2019 in rates of alcohol-associated liver disease for 15-19-year-olds by country.

| **North America** | | | |
| --- | --- | --- | --- |
| **Country** | **Direction of trend** | **Annual percentage change 2000-2019** | **P-value** |
| Canada | Stable | 0.25 | <0.001 |
| The United States of America | Decrease | -1.85 | <0.001 |
| **Other Anglosphere Countries** | | | |
| **Country** | **Direction of trend** | **Annual percentage change 2000-2019** | **P-value** |
| Australia | Stable | -0.14 | <0.001 |
| Ireland | Increase | 0.73 | <0.001 |
| New Zealand | Stable | 0.01 | 0.875 |
| The United Kingdom | Stable | 0.07 | 0.09 |
| **Mainland Europe** | | | |
| **Country** | **Direction of trend** | **Annual percentage change 2000-2019** | **P-value** |
| Austria | Decrease | -0.86 | <0.001 |
| Belgium | Increase | 0.83 | <0.001 |
| Estonia | Increase | 1.26 | <0.001 |
| Finland | Stable | 0.47 | <0.001 |
| Germany | Stable | 0.03 | 0.608 |
| Iceland | Increase | 0.92 | <0.001 |
| Lithuania | Increase | 1.54 | <0.001 |
| The Netherlands | Stable | 0.22 | <0.001 |
| Norway | Increase | 0.77 | <0.001 |
| Portugal | Decrease | -1.93 | <0.001 |
| Spain | Decrease | -0.72 | <0.001 |
| Sweden | Decrease | -0.57 | <0.001 |
| Switzerland | Increase | 1.19 | <0.001 |

*Note: Direction of trend based on 10% threshold rule, using annual percentage change multiplied by 19 years of data.*

## **Supplementary Table 4.** Summary of data extraction for n = 9 records with time spans of 5 years or less in the period of interest (2005 onwards).

| First author, publication year OR Data Summary | Country | Ages of interest | Study Time Period | Harm Measurement | Data Sources/Origins |
| --- | --- | --- | --- | --- | --- |
| Bouthoorn, 2011(91) | The Netherlands | 11-17 | 2007-2009 | Admissions to a department of paediatrics with a BAC>0 g/l and admitted due to impaired consciousness. | The NSCK – A national registration system for paediatric departments of Dutch hospitals. |
| Conway, 2010(89) | The UK: Scotland | 15-19 | 2005-2006 | Hospitalisation cases of inpatient care for adolescents with a diagnosis of facial injuries and a relevant supplementary alcohol-related diagnosis, according to ICD-10. | The Scottish Morbidity Record SMR01 (for general acute specialities), from the Information Services Division of the National Health Service, National Services Scotland. |
| Naeger, 2017(83) | America | 12-14  15-17 | 2010-2013 | Emergency department (ED) presentations with an alcohol-related diagnosis, according to ICD-9 codes. | The Nationwide Emergency Department Sample, from the Agency for Healthcare Research and Quality and the Healthcare Cost and Utilisation Project. |
| O’Donnell, 2017^a^(55) | Australia (Western Australia) | 13-17 | 1990-2009 | Hospital admissions with alcohol-related injuries, according to ICD-10 or T codes. | Both private and public hospital admission data, with population data provided by the Australian Bureau of Statistics. |
| Van Hoof, 2011(92) | The Netherlands | 11-18 | 2007-2008 | Alcohol intoxication admissions into a hospital department of paediatrics with a BAC>0.0g/L | The Dutch Paediatric Surveillance System. |
| Online report titled: Alcohol-related ED visits & hospitalisations & their co-occurring drug-related, mental health, & injury conditions in the United States(86) | America | 12-20 | 2006-2010 | Statistics on ED visits with principal (first-listed) alcohol-related diagnoses. | The Nationwide Emergency Department Sample & the Nationwide Inpatient Sample, published by the National Institute on Alcohol Abuse & Alcoholism. |
| Van Roozendaal, 2023(93) | Belgium | 10-17 | 2015-2019 | Alcohol-related hospitalisations from ED visits via either a positive Blood Alcohol Concentration (BAC) or screening of ED triage logs/hospital charts. | Data from hospitals managed by the Hospital Network Antwerp and by the Helix Network. |
| Renny et al., 2024(31) | America | 12-14  15-17 | 2018-2019 | Numbers of alcohol-related emergency department visits for youth. | Data from the electronic health records provided by the six hospitals within an urban healthcare system. Alcohol-related visits determined using ICD-10-CM codes. |
| Ball et al., 2024(30) | America | 12-21 | 2016-2019 | Numbers of alcohol-related emergency department visits and inpatient/observation visits of patients. | The Paediatric Health Information System (PHIS) database. The PHIS collects information from 47 tertiary care children’s hospitals within America. Data from 39 hospitals across 24 states are included in these results. Diagnosis codes are recorded via ICD-10-CM codes. |

*^a^Source has been split into two records to reflect differing results for separate countries (Australia and the UK). While the overall time period (1990-2009) spans 20 years, the number of years within the period of interest is 5 years.*

## **Supplementary Table 5.** Specific harm measurements for n = 41 sources.

| First author, publication year OR Data Summary | Specific Harm Measurement |
| --- | --- |
| Trefan, 2019(88) | Children (10-17 years) admitted to hospital, with International Classification of Diseases (ICD)-10 codes used to identify alcohol-related admissions. This was the presence of any of the following codes in the first three coding positions, or in the fourth if the first three only contained R or Z codes (excepting R78.0, Z50.2, Z71.4, Z72.1):  E24.4, E51.2, F10.0, F10.1, F10.2, F10.3, F10.4, F10.5, F10.6, 10.7, F10.8, F10.9, G31.2, G40.5, G62.1, G72.1, I42.6, K29.2, K70.0, K70.1, K70.2, K70.3, K70.4, K70.9, K85.2, K86.0, O35.4, R78.0, T51.0 (Excl.: acute alcohol intoxication or "hangover" effects (F10.0), drunkenness (F10.0), pathological alcohol intoxication (F10.0)), X45.0, X45.1, X45.2, X45.4, X45.5, X45.6, X45.8, X45.9, X65.0, X65.1, X65.2, X65.4, X65.5, X65.6, X65.8, X65.9, Y15.0, Y15.2, Y15.4, Y15.8, Y15.9, Y90.0, Y90.1, Y90.2, Y90.3, Y90.4, Y90.5, Y90.6, Y90.7, Y90.8, Y90.9, Y91.0, Y91.1, Y91.2, Y91.3, Y91.9, Z50.2, Z71.4, Z72.1 |
| Green, 2017(90) | Inclusion criteria:   1. Only finished episodes eligible. Non-elective admissions, elective admissions expected to be an overnight stay, day case, and maternity admissions were included. 2. Admissions with an age outside 0-120 or where sex not recorded as male/female excluded. 3. Admissions without an English residence postcode, unknown postcode, or no fixed abode excluded. 4. Alcohol-related admissions based on Public Health England’s ‘Narrow’ measure. Four outcome measures of alcohol-related harm were used (Current review focuses on acute conditions wholly attributable to alcohol), based on ICD-10 codes below. Only emergency admissions were considered for acute conditions.   Acute conditions wholly attributable to alcohol consumption ICD-10 codes:  F10.0, R78.0, T51, X45, X65, Y15, Y90, Y91 |
| Mitra, 2023(62) | Inclusion criteria:   1. Patients presenting to The Alfred Emergency and Trauma Centre. Only primary presentations were included. Inter-hospital transfers & patients without a BAC measured were excluded. 2. Inclusion in the Trauma Registry (AHTR). All major trauma admissions (Injury Severity Score > 12), all requiring admissions for over 72 hours, all trauma ICU admissions, and all deaths after injury included. 3. Patients aged 16-19 at time of injury between 1 January 2008 and 31 December 2019. 4. Patients had a BAC greater than 0.00g/100ml. |
| Moise, 2019(61) | Inclusion criteria:   - Identified in the Illinois State Trauma Registry following presentation to Level I and Level II trauma centres. For repeat victims, one random hospitalisation per year per person included. - Patients with a Chicago home address & zip code - Positive BAC (>0.0g/100ml). Includes encounters for those who died with or without resuscitation and those dead on arrival. Any missing data (approximately 20%) treated as negative BAC. - Patients with an unintentional injury principal diagnosis e-code according to the following ICD-9 codes:   E800-E869, E880-E929, E920 (.0-.9), E830 (.0-.9), E832 (.0-.9), E910 (.0-.9), E880.0-E886.9, E888, E890.0-E899, E924 (.0-.9), E922 (.0-.3, .8,.9), E919 (.0-.9), E810-E819 (.0-.9), E900.0-E909, E928  (.0-.9), E927, E850.0-E869.9, E916-E917.9, E846-E848, E914-E915, E918, E921 (.0-.9), E922 (.4-.5), E923(.0-.9)E925.0-E926.9, E928 (.3, .8), E929 (.0-.5, .8), E887, E928.9, E929.9 |
| Ngo, 2018(59) | Students who visited the university hospital emergency department (ED) during six academic years from 2009-10 to 2014-15. Visits due to alcohol intoxication were identified using the ICD-9 codes 305.0 and 303.0, which are documented on the patient’s unique EMR number. |
| O’Donnell, 2017(55) | Hospital admissions data from England and Western Australia (This source was split into two records for the current review). Inclusion criteria:   - Western Australia (WA) included injury admissions for those aged 13-17 to both private and public hospitals. England included injury admissions for those aged 13-17 to the National Health Service in England. - Patients admitted through the hospital’s admissions process for a period of care due to a sudden health issue. Planned or scheduled admissions were excluded. - ICD-10 codes for alcohol-related admissions included: F10, E24.4, G31.2, G62.1, G72.1 I42.1, K29.2, K70, K85.2, K86.0, R78.0, T50.6, T51, X45, X65, Y15, Y91, Z50.2, Z71.4, and Z72.1 - ICD-10 codes for injury (intentional or unintentional) included: Y04, Y05, X85-Y03, Y08-Y09, T74, T73, Y06, Y07, X60-X69, X70-X84, Z91.5, Y20-Y34, Z04.5, Z04.8, V01-V99 and W00-X59. |
| Panken, 2022(71) | Inclusion criteria:   - Patients older than 10 years of age - Presentation to either University Hospital Leuven (UZL) or Heilig Hart Leuven (HHL) between 1 January 2008 to 31 December 2019. If there was a repeat admission by the same patient, each visit was considered as a separate case. - BAC equal or higher than 0.1g/L |
| Sims, 2021(63) | Alcohol-related ED presentations identified via ICD-10-AM codes, symptom codes, or diagnosis discharge text:   - ICD-10-AM Codes: E24.4, E52, F10, G31.2, G40.5, G62.1, G72.1, I42.6, K29.2, K70, K85, K85.2, K86.0, O35.4, O99.3, P04.3, Q86.0, Q87.18, R78.0, T50.6, T51, X45, X65, Y15, Y90, Z71.4, Z72.1 - Symptom codes: E0000, EA000, EB000, ECA00 - Discharge text: Containing the words ‘alcohol’ or ‘intoxicated’ and presenting complain text containing the words ‘alcohol’ or ‘intoxicated’ |
| Tyrrell, 2018(67) | Inclusion criteria:   - Cohort registered with a Hospital Episode Statistics (HES)- Office for National Statistics (ONS) linked Clinical Practice Research Datalink (CPRD) practice aged between 10-24 years between 1 April 1998 and 31 March 2014 - Outcome of poisoning event occurs within the study period and recorded. Repeat poisoning events for the same individual were included. - For mortality data, only primary cause of death codes were included. - Poisonings and their intent were identified via ICD-10 codes and Read Codes in CPRD data. All poisoning substances recorded for an event were included.   ICD-10 Codes:  T36–65, X40–49, X60–69, Y10–19, and Y90–91. Excluding venomous animal and food poisonings  (T61, T63, and T64).  Read Codes are unavailable. |
| Wicki, 2020(73) | Hospitalisation data with alcohol intoxication diagnosed via F10.0, F10.1 and T51.0 ICD-10 codes. Primary and secondary diagnoses were considered. |
| Online database titled: Statistical database, diagnoses(74) | Parameters chosen on database:   - Type of care: In-patient and/or specialised open care (includes both hospital admissions and outpatient non-primary care, such as hospital-based ED visits) - Annual or monthly data: Annual data - Principal diagnoses: Alcohol Index* - Country of residence: Entire Sweden - Age: 10-14 years and 15-19 years - Sex: Both sexes - Measure: Number of patients, per 100,000 inhabitants, and age-standardised numbers 2022 - Years: 2008-2022   *The following ICD-10 codes are included in the Alcohol Index:  E24.4, F10, G31.2, G62.1, G72.1, I42.6, K29.2, K70.0-K70.9, K85.2, K86.0, O35.4, P04.3, Q86.0, T51.0-T51.9, Y90.1-Y90.9, Y91.1-Y91.9, Z50.2, Z71 |
| Downloadable dataset, page titled: Alcohol-related harm data(70) | Parameters chosen on dataset:   - Age group: 0-14 and 15-24 - District health board: New Zealand - Time period: 1 year - Period: 2005-2019   ICD-10 codes to define wholly attributable hospitalisations using principal diagnosis:  E24.4, F10, G31.2, G62.1, G72.1, I42.6, K29.2, K70, K86.0, T51.0, T51.1, T51.9, Q86.0, R78.0, K85.2 |
| Online database titled: Diagnostic data of the hospitals starting from 2000(72) | Patients who were discharged from inpatient treatment in a hospital in a reporting year, diagnosis of F10.0 according to ICD-10. |
| Online database titled: Local Alcohol Profiles for England(64) | Admissions to hospitals for under-18’s where the primary or any secondary diagnosis is an alcohol-specific, wholly attributable condition. Identification via ICD-10 codes – however cannot identify which specific ones used. |
| Downloadable dataset, page titled: Alcohol related hospital statistics(66) | Parameters chosen on dataset:   - Condition: All alcohol conditions - SMR type: Combined - Year: 2005/06 – 2018/19 - Gender: Age-group (5-yr), Age-group (5-yr) – male, Age-group (5-yr) – female - Age: Under 15 years and 15-19 years   ICD-10 codes for alcohol-related conditions:  F10.0 - F10.9, K70.0 - K70.4, K70.9, E51.2, G31.2, K85.2, K86.0, T51.0, T51.1, T51.9, I42.6, K29.2, E24.4, E51.2, G31.2, G62.1, G72.1, O35.4, P04.3, Q86.0, R78.0, X45, X65, Y15, Y57.3, Y90, Y91, Z50.2, Z71.4, Z72.1 |
| Online report titled: Alcohol Market, Consumption and Harms in Estonia – Yearbook (Alkoholi turg, tarbimine ja kahjud Eestis – Aastaraamat)(29-34) | Data presents visits paid by the Estonian Health Insurance Fund and the Ministry of Social Affairs – visits paid by people themselves are not included. Alcohol-related diseases identified via the following ICD-10 codes: F10.0, F10.3-10.9, F10.1, F10.2, F10, K29.2, K70-70.99, T51-T51.99 |
| Online report titled: Mortality Attributable to Alcohol in Spain (Mortalidad Atribuible al Alcohol en España)(50) | Excessive drinker: during the last year, consumed ≥60g of pure alcohol (men) or ≥40g of pure alcohol (women) daily.  Light/Moderate drinker: During the last year have consumed <60g of pure alcohol (men) or <40g of pure alcohol (women) daily. |
| Online report titled: Alcohol Consumption, Alcohol-related Harm and Alcohol Policy in Ireland(65) | Discharges from all acute Irish hospitals, excluding ED data. Wholly alcohol-attributable conditions identified via ICD-10 codes as follows:  ICD-10 codes for acute conditions: F10.0, T51.0, T51.1, T51.2, T51.3, T51.8, T51.9, X45, X65, Y15, R78.0, Y90.0-Y90.9, Y91.0-Y91.9  ICD-10 codes for chronic conditions: E24.4, G31.2, G62.1, G72.1, I42.6, K29.2, K70.0, K70.1, K70.2, K70.3, K70.4, K70.9, K86.0, K85.2  ICD-10 codes for other conditions: F10.1, F10.2, F10.3, F10.4, F10.5, F10.6, F10.7, F10.8, F10.9, Z50.2, Z71.4, Z72.1, Z86.41 |
| Downloadable dataset, page titled: Hospital Discharges from Acute Hospitals (Spitalsentlassungen aus Krankenanstalten)(51) | Parameters chosen on dataset:   - Year of discharge (“BERJ”): 2005 to 2019 - Sex (“GESCHLECHT”): Male and female (“1” and “2”) - Age (“ALTER”): Up to 14 years old (“1”) - Diagnosis (“DIAG”): ICD-10 code F10 (“025”) |
| Smith, 2023(56) | Data included wholly or partially alcohol-attributable conditions based on the ICD-10 codes below. Hospitalisations and ED visits were identified based on most responsible diagnosis code, except for injuries and motor vehicle accidents which were included if any diagnosis code was alcohol related.  ICD-10 codes for wholly attributable conditions:  E24.4, F10.0, F10.3 – F10.9, F10.1, F10.2, G31.2, G62.1, G72.1, I42.6, K29.2, K85.2, K86.0, T51 (External X45, Y15), T51 (External X65)  ICD-10 codes for partially attributable conditions:  A15 - A19, B20 - B24, Z21, J09 - J22, C00 – C05, C08 – C10, C12 – C14, D00.0, C15, D00.1 (portional only), C18 – C21, D01.0 – D01.4, C22, D01.5, C25, D01.7, C32, D02.0, C50, D05, E11, E13, E14, G40, G41, I10 – I15, I20 – I25, I47 - I49, I60 - I62, I69.0 – I69.2, I63 – I67, I69.3 – I69.4, I85, K70, K74, K85.0 – K85.1, K85.8 – K85.9, K86.1 – K86.9, External V1* & Y85.0, External W00-W19 & Y30, External W65-W74, External X00-X09 & Y26, T36 – T50, T52 – T65, T96 – T97 (External X40 – X44, X46 – X49, Y10 – Y14, Y16 – Y19), External V2*, W20-W64, W75-W84, X10 - X33, Y20, Y22 -Y25, Y27 - Y29, Y31 - Y34, Y85.9, Y86, Y87.2, Y89.9, T36 – T50, T52 – T65, T96 – T97, External X60 – X64 & X66 – X69, External X70 – X84 & Y87.0, External X85 – Y09 & Y87.1, External Y35 & Y89.0 |
| Myran, 2019(60) | An ED visit entirely caused by alcohol, with alcohol being listed as a main reason or having contributed to the ED visits, according to ICD-10 codes as follows:  F10.0-F10.9, E24.4, G31.2, G62.1, G72.1, I42.6, K29.2, K70.0-K70.4, K70.9, K85.2, K86.0, P04.3, Q86.0, O35.4, R78.0, T51.0, T51.9, X45, X65, Y15 |
| White, 2018(84) | Hospital-based ED visit identified as being caused by either acute or chronic alcohol consumption, with cases included if the diagnosis code or external causes of injury and poisoning (E-codes) noted an alcohol related code.  ICD-9 acute alcohol consumption codes: 303.0, 305.0, 790.3, 980, E860  ICD-9 chronic alcohol consumption codes: 291.0- 291.5, 291.8, 291.9, 303.9, 357.5, 425.5, 535.3, 571.0, 571.1, 571.2, 571.3, 571.4, 571.6, 571.8, 572.3, 571.5, 571.9 |
| Online report titled: Trends in alcohol-related morbidity among community hospital discharges(85) | Definitions of alcohol-related diagnoses are made based upon the below ICD-9 codes. The first-listed diagnosis is the principal diagnosis.  291.0, 291.1, 291.2, 291.3, 291.4, 291.5, 291.8, 291.9, 303.0, 303.9, 357.5, 425.5, 535.3, 305.0, 571.0-571.6, 571.8, 571.9, 572.3, 790.3, 980, E860 |
| Online database titled: Hospital admissions and patients (Ziekenhuisopnamen en -patiënten)(52) | Admissions include clinical admissions, day admissions, long-term observations, and the total number of hospital admissions in general, academic and two categorical hospitals (eye hospital and cancer clinic). Admissions are counted if the discharge date falls in the calendar year in question. Clinical recordings refer to a stay in a department designed for nursing, for which one or more nursing days are registered. Day recordings refer to a form of nursing lasting a number of hours in a department set up for day care, generally foreseeable and necessary in connection with a specialist medical examination or treatment taking place on the same day. Observation refers to a long-term observation without an overnight stay – an unplanned form of nursing of at least four consecutive hours with the aim of observing a patient.  Diagnoses were registered according to ICD-10 codes (or converted from ICD-9 codes to ICD-10). The principal diagnosis is the diagnosis considered to be the main reason for hospital admission at discharge. For the current review, data focused specifically on rates of ICD-10 code F10. |
| Online database titled: Patients in hospitals by age class, sex and diagnostic group (Patients dans les hôpitaux selon la classe l'âge, le sexe et le groupe de diagnostic)(53) | Parameters chosen on dataset:   - Observation Unit (Beobachtungseinheit): Inpatient cases (stationäre fälle) - Age group (Altersklasse): 10-14 years and 15-19 years (10-14 Jahre, 15-19 Jahre) - Gender (Geschlecht): Gender – Total (Geschlecht – Total) - Diagnostic Group (Diagnosegruppe): Mental and behavioural disorders caused by alcohol (Psychische und verhaltensstörungen durch alcohol) - Year (Jahr): 2005-2019 |
| Online database titled: Canadian Substance Use Costs and Harms(57) | Inpatient hospitalisations refer to admissions to a hospital bed in an acute care hospital for at least one night due to an alcohol-attributable health condition. An ED visit refers to an ED hospital visit for an alcohol-attributable health condition. The data does not include hospitalisations & ED visits in Quebec. It is noted that ED visits attributable to alcohol are underestimated by roughly 3% for all years. There is also likely to be an underestimation in hospitalisations for the Ontario & Manitoba provinces – as both do not include hospitalisations recorded in the Ontario Mental Health Recording System, because the database does not use the ICD-10 system.  Alcohol-related harms are measured according to the ICD-10 coding system, however specific codes used cannot be found. |
| Online database titled: Australian alcohol-attributable harm visualisation tool(68) | Health conditions related to the impacts of maternal consumption on the foetus have been excluded from the analysis. Records where sex was either ‘other’ or missing were also excluded. Wholly alcohol-attributable conditions were identified via the following ICD-10 codes:  I42.6, K29.2, K70, K86.0, E24.4, T51.0, T51.1, T51.9, X45, Y15, X65, F10.0-F10.1, F10.2, F10.3-F10.9, G31.2, G62.1, G72.1 |
| Downloadable dataset, page titled: Alcohol-related injury: Hospitalisations and deaths, 2019-2020(69) | The term ‘alcohol-related’ refers to all cases, regardless of the extent to which alcohol may have contributed to the injury event. Diagnosis is based on ICD-10 codes; however, data quality is noted to depend on the extent to which hospital staff record the involvement of alcohol.  Hospitalisation inclusion criteria:   - A principal diagnosis of injury AND an additional in-scope diagnosis relating to alcohol use OR an external cause of injury related to alcohol use. - A principal diagnosis of acute alcohol intoxication AND an additional diagnosis of injury. - ED presentations are excluded.   ICD-10 codes for defining injury: S00–T75 and T79  ICD-10 codes for defining alcohol-related conditions: F10.0, F10.1, F10.2, R78.0, T51.0, T51.9, X45, X65, Y15, Y90, Z72.1 |
| Downloadable dataset, page titled: National estimates of drug-related emergency department visits, 2004-2011 – All visits(87) | Data is compiled by the Drug Abuse Warning Network (DAWN). Noted that DAWN relies on a comprehensive drug vocabulary and classification system called the Drug Reference Vocabulary (DRV). This is a system of tables that DAWN uses to record, code, and classify reportable substances. The foundation of the DRV is the Multum Lexicon. However, cannot find any more specific information on this system. |
| Online database titled: Prevalence – number of ill people(54) | Prevalence rates refer to the number of persons with at least 1 registered diagnosis per year in health care institutions (including hospitals, ED’s, primary health care) and on death certificates, according to ICD-10 codes. For the current review, focused on the ICD-10 code of F10. |
| Bouthoorn, 2011(91) | Eligibility criteria:   - Patients admitted to a department of paediatrics in 2007, 2008, or 2009. - Patients aged between 11-17 years. - Patients with a BAC > 0.0g/L. - Patients admitted due to impaired consciousness |
| Conway, 2010(89) | Cases of inpatient care for patients with a diagnosis of facial injuries with relevant supplementary alcohol-related diagnosis, according to ICD-10 codes.  ICD-10 facial injury codes: S00.0, S00.1 (Excludes S05.1), S00.2 (Excludes S05.0), S00.3, S00.4, S00.5, S00.7, S00.8, S00.9, S01.0 (Excludes S08.0), S01.1, S01.2, S01.3, S01.4, S01.5 (Excludes S03.2 & S02.5), S01.7, S01.8, S01.9, S02.2, S02.3 (Excludes S02.8 & S02.1), S02.4, S02.5, S02.6, S02.7, S02.8 (Excludes S02.3 & S02.1), S02.9, S03.0, S03.1, S03.2, S03.3, S03.4, S03.5, S04.0, S04.1, S04.2, S04.3, S04.4, S04.5, S04.9, S05.1 (Excludes S00.1 & S00.1), S05.4 (Excludes H05.5), S05.8, S05.9, S07.0, S08.0, S08.1, S08.8, S08.9 (Excludes S18), S09.0 (Excludes S06.- & S15.-), S09.1, S09.2, S09.7, S09.8, S09.9, S10.7, S10.8, S10.9, S15.0, S15.2, S15.3, S15.7, S15.8, S15.9, S17.8, S17.9, S19.7, S19.8, S19.9  ICD-10 alcohol-related diagnosis codes: F10.0, F10.1, R78.0, T51, X45, X65, Y15, Y90, Y91, E52, G312, G621, G721, I426, K292, K70, K860, Y573, Z502, Z714, Z721 |
| Naeger, 2017(83) | ED visits for patients aged 12-20 with one of the following ICD-9 codes in any diagnostic or E-code field were included:  303.00, 303.01, 303.02, 305.00, 305.01, 305.02, 790.3, E860.0, 980.0, E860.0, 303.00, 303.01, 303.02, 305.00, 305.01, 305.02, 790.3 |
| Van Hoof, 2011(92) | Cases of adolescents aged 0-18 years old with a BAC > 0.0g/L were included. |
| Online report titled: Alcohol-related ED visits & hospitalisations & their co-occurring drug-related, mental health, & injury conditions in the United States(86) | The first-listed diagnosis is the “code for the diagnosis, condition, problem or other reason for encounter/visit shown in the medical record to be chiefly responsible for the services provided.”  Diagnosis of ED visits are based on the following ICD-9 codes:  291.0-291.5, 291.8, 291.9, 303.0, 303.9, 357.5, 425.5, 535.3, 305.0, 571.0-571.6, 571.8, 571.9, 572.3, 790.3, 980, E860. |
| Van Roozendaal, 2023(93) | Adolescents (aged 10-17) who experienced alcohol-related ED admissions were included. Patients were identified by either of the following:   - Clinical biologists working at hospitals identifying patients with a positive BAC (>0.03g/L or > 0.1g/L depending on the laboratory) - Identification using screening of ED triage logs and hospital charts for alcohol/intoxication-related words.   Patients were excluded if, based on their history, they did not drink alcohol and those with a negative BAC. |
| Stafström, 2023(18) | Patients diagnosed either through in-patient care (hospital admissions) and/or through specialised out-patient care (treated by a medical doctor, visits to ED, etc). Article did not specify which method was chosen.  Alcohol-related diagnoses are based on the following ICD-10 codes:  E24.4, F10, G31.2, G62.1, G72.1, I42.6, K29.2, K70.0-K70.9, K85.2, K86.0, O35.4, P04.3, Q86.0, T51.0-T51.9, Y90.1-Y90.9, Y91.1-Y91.9, Z50.2, Z71.4. |
| Online report titled: Yearbook of Alcohol and Drug Statistics (Päihdetilastollinen vuosikirja; Alkoholi ja huumeet)(35-49) | Hospital inpatient care periods with a primary diagnosis of alcohol-related diseases, according to ICD codes, as follows:  ICD-9 Codes: 2650A, 291, 303, 3050A, 3575A, 4255A, 5307A, 5353A, 5710A-5713X, 5770D-5770F, 5771C-5771D, 6484A, 7607A, 980.  ICD-10 Codes: F10, T51, K70, K85.2, K86.0, I42.6, K29.2, E51.2, E52, E24.4, G31.2, G40.51, G62.1, G72.1, P04.3, Q86.0, O35.4, Z50.2, Z71.4, Z72.1, R78.0, X45. |
| Renny et al., 2024(31) | Emergency department visits identified as being alcohol-related according to the following  ICD-10-CM codes:  F10.1X, F10.2X, F10.9X |
| Ball et al., 2024(30) | Alcohol-related emergency department visits or inpatient/observation visits of patients. These were identified using ICD-10-CM codes as follows:  Poisoning codes: T51.8X, T51.9X  Dependence codes: F10.2X  Use codes: F10.1X, F10.9X |
| Danpanichkul et al., 2024(29) | Cases of chronic liver disease and cirrhosis from alcohol consumption were identified using ICD-10 codes as follows:  Liver cancer due to alcohol use: C22.0-C22.1, C22.3-C22.8, and a proportion of C22.9 reflecting primary liver cancer.  Cirrhosis and other chronic liver diseases due to alcohol use: K70-70.9 |

## **Supplementary Table 6.** Quality assessment results table for n = 41 sources.

| **First Author, Publication Year  OR Data Summary** | **First Reviewer** | **Second Reviewer** | **Item 1** | **Item 2** | **Item 3** | **Item 4** | **Item 5** | **Item 6** | **Item 7** | **Item 8** | **Item 9** | **Item 10** | **Summary Item** |
| --- | --- | --- | --- | --- | --- | --- | --- | --- | --- | --- | --- | --- | --- |
| Bouthoorn, 2011 | EV | NT | Low Risk | High Risk | High Risk | Low Risk | High Risk | Low Risk | Low Risk | High Risk | Low Risk | Low Risk | **Moderate risk** |
| Trefan, 2019 | EV | ML | Low Risk | Low Risk | Low Risk | Low Risk | Low Risk | Low Risk | Low Risk | Low Risk | Low Risk | Low Risk | **Low risk** |
| Conway, 2010 | ML | NT | Low Risk | Low Risk | Low Risk | Low Risk | Low Risk | Low Risk | Low Risk | Low Risk | Low Risk | Low Risk | **Low risk** |
| Green, 2017 | EV | ML | Low Risk | Low Risk | Low Risk | Low Risk | Low Risk | Low Risk | Low Risk | Low Risk | Low Risk | Low Risk | **Low risk** |
| Mitra, 2023 | ML | EV | Low risk | High Risk | Low Risk | Low Risk | Low Risk | Low Risk | Low Risk | Low Risk | Low Risk | Low Risk | **Low risk** |
| Moise, 2019 | EV | ML | Low risk | High Risk | Low Risk | Low Risk | Low Risk | Low Risk | Low Risk | Low Risk | Low Risk | Low Risk | **Low risk** |
| Naeger, 2017 | ML | EV | Low Risk | Low Risk | Low Risk | Low Risk | Low Risk | Low Risk | Low Risk | Low Risk | Low Risk | Low Risk | **Low risk** |
| Ngo, 2018 | NT | ML | High Risk | Low Risk | Low Risk | Low Risk | Low Risk | Low Risk | Low Risk | Low Risk | Low Risk | Low Risk | **Low risk** |
| O'Donnell, 2017 | ML | EV | High Risk | Low Risk | Low Risk | Low Risk | Low Risk | Low Risk | Low Risk | Low Risk | Low Risk | Low Risk | **Low risk** |
| Panken, 2022 | NT | ML | High Risk | Low Risk | High Risk | Low Risk | Low Risk | Low Risk | Low Risk | High Risk | Low Risk | Low Risk | **Moderate risk** |
| Sims, 2021 | ML | NT | High Risk | Low Risk | Low Risk | Low Risk | Low Risk | Low Risk | Low Risk | High Risk | Low Risk | Low Risk | **Low risk** |
| Tyrrell, 2018 | EV | ML | Low Risk | Low Risk | Low Risk | Low Risk | Low Risk | Low Risk | Low Risk | Low Risk | Low Risk | Low Risk | **Low risk** |
| van Hoof, 2011 | ML | NT | Low Risk | Low Risk | Low Risk | High Risk | High Risk | Low Risk | Low Risk | High Risk | Low Risk | Low Risk | **Moderate risk** |
| Wicki, 2020 | ML | NT | Low Risk | Low Risk | Low Risk | Low Risk | Low Risk | High Risk | Low Risk | Low Risk | Low Risk | Low Risk | **Low risk** |
| Online database titled: Statistical Database, Diagnoses | NT | ML | Low Risk | Low Risk | Low Risk | Low Risk | Low Risk | Low Risk | Low Risk | Low Risk | Low Risk | Low Risk | **Low risk** |
| Downloadable dataset, page titled: Alcohol-related harm data | ML | NT | Low Risk | Low Risk | Low Risk | Low Risk | Low Risk | Low Risk | Low Risk | Low Risk | Low Risk | Low Risk | **Low risk** |
| Online database titled: Diagnostic data of the hospitals starting from 2000 | ML | NT | Low Risk | Low Risk | Low Risk | Low Risk | Low Risk | Low Risk | Low Risk | Low Risk | Low Risk | Low Risk | **Low risk** |
| Online database titled: Local Alcohol Profiles for England | NT | EV | Low Risk | Low Risk | Low Risk | Low Risk | Low Risk | Low Risk | Low Risk | Low Risk | Low Risk | Low Risk | **Low risk** |
| Downloadable dataset, page titled: Alcohol related hospital statistics | NT | EV | Low Risk | Low Risk | Low Risk | Low Risk | Low Risk | Low Risk | Low Risk | Low Risk | Low Risk | Low Risk | **Low risk** |
| Online report titled: Alcohol Market, Consumption and Harms in Estonia – Yearbook (Alkoholi turg, tarbimine ja kahjud Eestis – Aastaraamat) | EV | ML | Low Risk | High Risk | Low Risk | Low Risk | Low Risk | Low Risk | Low Risk | Low Risk | Low Risk | High Risk | **Low risk** |
| Online report titled: Mortality Attributable to Alcohol in Spain (Mortalidad Atribuible al Alcohol en España) | NT | EV | Low Risk | Low Risk | Low Risk | Low Risk | Low Risk | Low Risk | Low Risk | Low Risk | Low Risk | Low Risk | **Low risk** |
| Online report titled: Alcohol Consumption, Alcohol-related Harm and Alcohol Policy in Ireland | ML | NT | Low Risk | Low Risk | Low Risk | Low Risk | Low Risk | Low Risk | Low Risk | Low Risk | Low Risk | High Risk | **Low risk** |
| Downloadable dataset, page titled: Hospital Discharges from Acute Hospitals (Spitalsentlassungen aus Krankenanstalten) | NT | ML | Low Risk | Low Risk | Low Risk | Low Risk | Low Risk | Low Risk | Low Risk | Low Risk | Low Risk | High Risk | **Low risk** |
| Smith, 2023 | NT | EV | High Risk | Low Risk | Low Risk | Low Risk | High Risk | Low Risk | Low Risk | Low Risk | Low Risk | Low Risk | **Low risk** |
| Myran, 2019 | NT | EV | High Risk | Low Risk | Low Risk | Low Risk | Low Risk | Low Risk | Low Risk | Low Risk | Low Risk | Low Risk | **Low risk** |
| White, 2018 | EV | NT | Low Risk | Low Risk | High Risk | Low Risk | Low Risk | Low Risk | Low Risk | Low Risk | Low Risk | Low Risk | **Low risk** |
| Online report titled: Trends in alcohol-related morbidity among community hospital discharges | EV | ML | Low Risk | Low Risk | Low Risk | Low Risk | Low Risk | Low Risk | Low Risk | Low Risk | Low Risk | Low Risk | **Low risk** |
| Online report titled: Alcohol-related ED visits & hospitalisations & their co-occurring drug-related, mental health, & injury conditions in the United States | NT | EV | Low Risk | Low Risk | Low Risk | Low Risk | Low Risk | Low Risk | Low Risk | High Risk | Low Risk | Low Risk | **Low risk** |
| van Roozendaal, 2023 | EV | ML | High Risk | Low Risk | Low Risk | Low Risk | High Risk | Low Risk | Low Risk | High Risk | Low Risk | Low Risk | **Moderate risk** |
| Online database titled: Hospital admissions and patients (Ziekenhuisopnamen en -patiënten) | ML | NT | Low Risk | Low Risk | Low Risk | Low Risk | Low Risk | Low Risk | Low Risk | Low Risk | Low Risk | Low Risk | **Low risk** |
| Online database titled: Patients in hospitals by age class, sex and diagnostic group (Patients dans les hôpitaux selon la classe l’âge, le sexe et le groupe de diagnostic) | EV | NT | Low Risk | Low Risk | Low Risk | Low Risk | Low Risk | Low Risk | Low Risk | Low Risk | Low Risk | High Risk | **Low risk** |
| Online database titled: Canadian Substance Use Costs and Harms | NT | ML | Low Risk | Low Risk | Low Risk | Low Risk | Low Risk | Low Risk | Low Risk | Low Risk | Low Risk | Low Risk | **Low risk** |
| Online database titled: Australian alcohol-attributable harm visualisation tool | EV | ML | Low Risk | Low Risk | Low Risk | Low Risk | High Risk | Low Risk | Low Risk | Low Risk | Low Risk | Low Risk | **Low risk** |
| Downloadable dataset, page titled: Alcohol-related injury: hospitalisations and deaths, 2019-20 | ML | EV | Low Risk | Low Risk | Low Risk | Low Risk | Low Risk | Low Risk | Low Risk | Low Risk | Low Risk | High Risk | **Low risk** |
| Downloadable dataset, page titled: National estimates of drug-related emergency department visits, 2004-2011 – All visits | ML | EV | Low Risk | Low Risk | Low Risk | Low Risk | Low Risk | Low Risk | Low Risk | Low Risk | Low Risk | Low Risk | **Low risk** |
| Online database titled: Prevalence – number of ill people | EV | NT | Low Risk | Low Risk | Low Risk | Low Risk | Low Risk | Low Risk | Low Risk | Low Risk | Low Risk | Low Risk | **Low risk** |
| Stafström, 2024 | EV | NT | Low Risk | Low Risk | Low Risk | Low Risk | Low Risk | Low Risk | Low Risk | Low Risk | Low Risk | Low Risk | **Low risk** |
| Online report titled: Yearbook of Alcohol and Drug Statistics (Päihdetilastollinen vuosikirja; Alkoholi ja huumeet) | NT | EV | Low Risk | Low Risk | Low Risk | Low Risk | Low Risk | Low Risk | Low Risk | Low Risk | Low Risk | High Risk | **Low risk** |
| Renny et al., 2024 | EV | ML | High Risk | Low Risk | Low Risk | Low Risk | Low Risk | Low Risk | Low Risk | Low Risk | Low Risk | High Risk | **Low risk** |
| Ball et al., 2024 | EV | ML | Low Risk | High Risk | Low Risk | Low Risk | Low Risk | Low Risk | Low Risk | Low Risk | Low Risk | High Risk | **Low risk** |
| Danpanichkul et al., 2024 | EV | ML | Low Risk | Low Risk | Low Risk | Low Risk | Low Risk | Low Risk | Low Risk | High Risk | Low Risk | Low Risk | **Low risk** |

*Note: Low risk of bias if source had 0-2 items scoring high risk, moderate risk of bias if source had 3-4 items scoring high risk, high risk of bias if five or more items scoring high risk. Tool created by Hoy et al. (28)*

## **Supplementary Table 7.** Instances where source variables are included despite being outside the original eligibility criteria and other biases or limitations noted within included studies.

| First author, publication year OR Data Summary | Variable | Record measured variable | Other biases or limitations |
| --- | --- | --- | --- |
| Online database titled: Statistical database, diagnoses(74) | N/A |  | Source notes that results should be interpreted with caution, as changes may be due to improved reporting and not increases in people seeking care |
| Downloadable dataset, page titled: Alcohol-related harm data(70) | Population age | Ages 0-14 and 15-24 | Indicator does not include visits to the emergency department requiring less than 3 hours of care |
| Online database titled: Local Alcohol Profiles for England(64) | Population age | Age group of ‘under 18’s’ (no clarification given) | Does not include attendance at accident and emergency departments |
| Downloadable dataset, page titled: Alcohol-related hospital statistics(66) | Population age | One age group is ‘under 15’s’ (no clarification given) | Does not include attendances at accident and emergency that does not result in hospital admission |
| Online report titled: Alcohol Market, Consumption and Harms in Estonia – Yearbook (Alkoholi turg, tarbimine ja kahjud Eestis – Aastaraamat)(29-34) | Population age | Ages 16-20 | Note some consistency issues when comparing different yearly reports. |
| Online report titled: Mortality Attributable to Alcohol in Spain (Mortalidad Atribuible al Alcohol en España)(50) | Population age, Time period | Ages 15-34, first time period is 2001-2009 (being compared to 2010-2017) | Note very low numbers (using mortality data) |
| Online report titled: Alcohol consumption, alcohol-related harm and alcohol policy in Ireland(65) | Population age, Time period | Ages 0-17, first time point is 1995 (being compared to 2018) |  |
| Downloadable dataset, page titled: Hospital Discharges from Acute Hospitals (Spitalsentlassungen aus Krankenanstalten)(51) | Population age | Ages 0-14 |  |
| Smith, 2023(56) | Population age | Ages 15-34 | Data specific to Ontario (Canada). Alcohol-attributable trends are modelled; Alcohol causes are not specifically coded in the data, meaning trends could be influenced by other factors. |
| Myran, 2019(60) | N/A |  | Data specific to Ontario (Canada) |
| Online report titled: Trends in alcohol-related morbidity among community hospital discharges(85) | Population age | Ages 12-20 | Does not include federal hospitals, rehabilitation hospitals, or those with an average length of stay over 20 days. |
| Online report titled: Alcohol-related ED visits & hospitalisations & their co-occurring drug-related, mental health, & injury conditions in the United States(86) | Population age | Ages 12-20 |  |
| Van Roozendaal, 2023(93) | N/A |  | Data specific to Antwerp (Belgium). No mention in source regarding if BAC is routinely collected. Additionally, ED triage logs & hospital charts used for data, therefore intoxication could merely be suspected, not proven. |
| Online database titled: Hospital admissions and patients (Ziekenhuisopnamen en -patiënten)(52) | Population age | Ages 1-20 |  |
| Online database titled: Canadian substance use costs and harms(57) | Population age | Ages 0-14 | Does not include data from Quebec. Source notes that ED visits due to alcohol are underestimated by roughly 3% for all years. Also noted there is likely to be an underestimation in hospitalisation for Ontario & Manitoba provinces. |
| Online database titled: Australian alcohol-attributable harm visualisation tool(68) | Population age | Ages 15-34 |  |
| Downloadable dataset, page titled: Alcohol-related injury: hospitalisations and deaths, 2019-2020(69) | Population age | Ages 15-24 |  |
| Downloadable dataset, page titled: National estimates of drug-related emergency department visits, 2004-2011 – All visits(87) | Population age | Ages 18-20 and age group ‘under 21’s’ (no clarification given) |  |
| Stafström, 2023(16) | Time Period | Trend for entire period 2000-2021, no yearly data available |  |
| Renny et al., 2024(31) | N/A |  | Data is sourced from six hospitals across America and is not nationally representative. |
| Ball et al., 2024(30) | Population Age | Ages 12-21 |  |
| Danpanichkul et al., 2024(29) | Time Period | 2000-2019 |  |
| Online report titled: Yearbook of Alcohol and Drug Statistics (Päihdetilastollinen vuosikirja; Alkoholi ja huumeet)(35-49) | Population age | Ages 0-14 | Noting it is likely that reporting practices improved over the years. |
| Conway, 2010(89) | N/A |  | Study specifically looks at facial injuries related to alcohol. |
| Green, 2017(90) | N/A |  | No raw data available (results based on interpretation of figures reported). |
| Mitra, 2023(62) | N/A |  | Results focused on a single trauma centre in Victoria, Australia. |
| Moise, 2019(61) | N/A |  | Study focused in Chicago, America, and relies on trauma centre data only |
| Ngo, 2018(59) | N/A |  | Study focused on a single college emergency department in America |
| Panken, 2022(71) | N/A |  | Study focuses on two hospitals, both within a single student city in Belgium. Additionally, the harm measurement was completed only at the doctor’s discretion. |
| Sims, 2021(63) | N/A |  | Source notes that the data is most likely underestimated – ED presentations resulting in a discharge home are not included. Additionally, there are no mandatory requirements for ED’s to routinely collect alcohol presentation data. |
| Tyrrell, 2018(67) | N/A |  | Source notes that the data is likely an under-estimation of incidence – Only 57.8% of poisonings had a specific substance recorded. |
| Wicki, 2020(73) | N/A |  | Note that results used in review focus on the entirety of Switzerland but excluding the Canton of Vaud, therefore excluding the area impacted by introduced alcohol-related policy changes. |

*Note: N/A indicates there were no instances. Any sources not included in this table indicates there were no instances and no further biases or limitations noted from this source.*

## **Supplementary Table 8.** Alternative approaches to presenting harm trend results for n = 37 records with timespans over 5 years.

| **Approach** |  | **Increases** | **Decreases** | **Stable** | **Mixed** | **Total** | **Notes** |
| --- | --- | --- | --- | --- | --- | --- | --- |
| Results by region (current approach used) | North America | 1 | 4 | 2 | 4 | 11 |  |
|  | Other Anglosphere Countries | 0 | 8 | 1 | 4 | 13 |  |
|  | Mainland Europe | 3 | 4 | 2 | 4 | 13 |  |
| Results by record standard* (approach not chosen) | Gold-standard | 0 | 3 | 2 | 2 | 7 | This approach not chosen due to a lack of gold-standard studies, making it difficult to compare across countries |
|  | Near gold-standard | 2 | 7 | 1 | 4 | 14 |  |
|  | Standard | 2 | 5 | 1 | 6 | 14 |  |
|  | Sub-standard | 0 | 1 | 1 | 0 | 2 |  |
| Results by trend outcome (approach not chosen) |  | 4 | 16 | 5 | 12 | 37 | Each trend outcome has a variety of countries, harm magnitudes, measurement types. There is no clear unifier. Mixed results are usually sex-based differences. |
|  |  | **Same trend direction for males & females** | | **Differing trend direction for males & females** | | **Total** |  |
| Reporting sex-specific trends only (n=19)  (approach not chosen) | North America | 3 | | 3 | | 6 | Differences due to males decreasing and females increasing (n=1) or males stable and females increasing (n=2). |
|  | Other Anglosphere Countries | 4 | | 4 | | 8 | Differences due to males consistently decreasing, females stable (n=3) or increasing (n=1). |
|  | Mainland Europe | 4 | | 1 | | 5 | Difference due to young males decreasing, older males & females increasing (n=1). |
|  | **Total** | 11 | | 8 | | 19 |  |

**Record standard was based on the four key variables that each record would ideally have to be considered a ‘gold-standard’ study. These are: reporting national statistics, data presented as rates (rather than numbers or proportions, for example), age-group within 10-19 years, and data presented over the correct time period (2005-2019). Gold-standard studies are those that fulfill all four of the key variables, near gold-standard fulfill three of the key variables, standard fulfill two of the key variables, and sub-standard fulfill less than two of these key variables.*

## **Supplementary Table 9.** Table reporting synthesis without meta-analysis (SWiM) reporting guidelines.

| **SWiM (58) reporting item** | **Item description** | **Page in manuscript where item is reported** |
| --- | --- | --- |
| *Methods* | | |
| **1** Grouping studies for synthesis | 1a) Provide a description of, and rationale for, the groups used in the synthesis (e.g., groupings of populations, interventions, outcomes, study design) | Page 7 (Results, Presentation of Results, first paragraph) & Supplementary Table 8 |
|  | 1b) Detail and provide rationale for any changes made subsequent to the protocol in the groups used in the synthesis | N/A – no changes made |
| **2** Describe the standardised metric and transformation methods used | Describe the standardised metric for each outcome. Explain why the metric(s) was chosen, and describe any methods used to transform the intervention effects, as reported in the study, to the standardised metric, citing any methodological guidance consulted | Page 5 (Methods, Synthesis of Results) |
| **3** Describe the synthesis methods | Describe and justify the methods used to synthesise the effects for each outcome when it was not possible to undertake a meta-analysis of effect estimates | Page 5 (Methods, Synthesis of Results) |
| **4** Criteria used to prioritise results for summary and synthesis | Where applicable, provide the criteria used, with supporting justification, to select the particular studies, or a particular study, for the main synthesis or to draw conclusions from the synthesis (e.g., based on study design, risk of bias assessments, directness in relation to the review question) | Page 6 (Results, Description of Included Studies, final paragraph) |
| **5** Investigation of heterogeneity in reported effects | State the method(s) used to examine heterogeneity in reported effects when it was not possible to undertake a meta-analysis of effect estimates and its extensions to investigate heterogeneity | Page 7 (Results, Presentation of Results, first paragraph) & Supplementary Table 8 |
| **6** Certainty of evidence | Describe the methods used to assess certainty of the synthesis findings | Page 6 (Results, Quality Assessment Results & Supplementary Table 6) |
| **7** Data presentation methods | Describe the graphical and tabular methods used to present the effects (e.g., tables, forest plots, harvest plots).  Specify key study characteristics (e.g., study design, risk of bias) used to order the studies, in the text and any tables or graphs, clearly referencing the studies included | Page 7 (Results, Presentation of results, second paragraph) |
| *Results* | | |
| **8** Reporting results | For each comparison and outcome, provide a description of the synthesised findings, and the certainty of the findings. Describe the result in language that is consistent with the question the synthesis addresses, and indicate which studies contribute to the synthesis | Page 7-9 (Results, trends in alcohol-related harm), Table 6 and Supplementary Table 10. |
| *Discussion* | | |
| **9** Limitations of the synthesis | Report the limitations of the synthesis methods used and/or the groupings used in the synthesis, and how these affect the conclusions that can be drawn in relation to the original review question | Page 9-11 (Discussion, Limitations) |

## **Supplementary Table 10.** Key results regarding trends in alcohol-related harms for n = 9 records with time spans of 5 years or less in the period of interest.

| Country | First Author, Publication Year  OR Data Summary | Direction of Trend | Proportion | Timespan | Measure |
| --- | --- | --- | --- | --- | --- |
| America | Naeger, 2017(83) | Decrease | 36% decline in rates for 12-14-year-old males.  43% decline in rates for 12-14-year-old females.  20% decline in rates for 15-17-year-old males.  16% decline in rates for 15-17-year-old females. | 2010-2013 | Rates of alcohol-only emergency department (ED) presentations per 100,000 |
| The UK: Scotland | Conway, 2010(89) | Stable | Stability in rates for 15-19-year-olds. | 2005-2006 | Rates of facial injury diagnoses with a concomitant alcohol-related diagnosis per 1000 persons. |
| The Netherlands | Bouthoorn, 2011(91)* | Increase | 80% increase in numbers for 11-17-year-olds. | 2007-2009 | Numbers of hospital admissions due to alcohol intoxication. |
| Australia | O’Donnell, 2017(55) | Increase | 46% increase in rates for 13-17-year-olds. | 1990-2009 | Rates of alcohol-related admissions per 10,000.  Note, data available for the entire period only. |
| The Netherlands | van Hoof, 2011(92)* | Increase | 13% increase in numbers for 11-18-year-olds. | 2007-2008 | Numbers of alcohol-related admissions. |
| America | Online report titled: Alcohol-related ED visits & hospitalisations & their co-occurring drug-related, mental health, & injury conditions in the United States(86) | Increase | 10% increase in rates for 12-20-year-olds. | 2006-2010 | Rates of principal alcohol-related ED visits per 10,000 population. |
| Belgium | van Roozendaal, 2023(93)* | Increase | 56% increase in rates for 10-17-year-olds. | 2015-2019 | Rates of alcohol-related ED visits per 10,000. |
| America | Renny et al., 2024(31) | Mixed | Stability for 12-14-year-olds  88.57% increase for 15-17-year-olds. | 2018-2019 | Numbers of alcohol-related ED visits compared to all ED visits. |
| America | Ball et al., 2024(30) | Stability | Stability in numbers for 12-21-year-olds | 2016-2019 | Numbers of alcohol-related ED or inpatient/observation visits. |

*^a^Source has been split into two records to reflect differing results for separate countries (Australia and the UK). While the overall time period (1990-2009) spans 20 years, the number of years within the period of interest is 5 years.*

**Indicates records that scored a moderate risk of bias according to Hoy et al. (28)*
